# The Effect of Vacancies on Grain Boundary Segregation in Ferromagnetic *fcc* Ni

**DOI:** 10.3390/nano10040691

**Published:** 2020-04-06

**Authors:** Martina Mazalová, Monika Všianská, Jana Pavlů, Mojmír Šob

**Affiliations:** 1 Department of Chemistry, Faculty of Science, Masaryk University, Kotlářská 2, CZ-611 37 Brno, Czech Republic; 394206@mail.muni.cz (M.M.); 230038@mail.muni.cz (M.V.); houserova@chemi.muni.cz (J.P.); 2 Institute of Physics of Materials, Academy of Sciences of the Czech Republic, Žižkova 22, CZ-616 62 Brno, Czech Republic; 3 Central European Institute of Technology, CEITEC MU, Masaryk University, Kamenice 753/5, CZ-625 00 Brno, Czech Republic

**Keywords:** *fcc* Ni, tilt Σ5(210) grain boundary, vacancy, Si and Al impurity, grain boundary energy, segregation energy, defects binding energies, magnetism

## Abstract

This work presents a comprehensive and detailed ab initio study of interactions between the tilt Σ5(210) grain boundary (GB), impurities X (X = Al, Si) and vacancies (Va) in ferromagnetic *fcc* nickel. To obtain reliable results, two methods of structure relaxation were employed: the automatic full relaxation and the finding of the minimum energy with respect to the lattice dimensions perpendicular to the GB plane and positions of atoms. Both methods provide comparable results. The analyses of the following phenomena are provided: the influence of the lattice defects on structural properties of material such as lattice parameters, the volume per atom, interlayer distances and atomic positions; the energies of formation of particular structures with respect to the standard element reference states; the stabilization/destabilization effects of impurities (in substitutional (s) as well as in tetragonal (iT) and octahedral (iO) interstitial positions) and of vacancies in both the bulk material and material with GBs; a possibility of recombination of Si^(i)^+Va defect to Si^(s)^ one with respect to the Va position; the total energy of formation of GB and Va; the binding energies between the lattice defects and their combinations; impurity segregation energies and the effect of Va on them; magnetic characteristics in the presence of impurities, vacancies and GBs. As there is very little experimental information on the interaction between impurities, vacancies and GBs in *fcc* nickel, most of the present results are theoretical predictions, which may motivate future experimental work.

## 1. Introduction

Various crystal defects such as impurities, vacancies (Va) and grain boundaries (GB) significantly affect material properties and are objects of both theoretical and applied research. Recent investigations deal with topics such as energetics of GB formation and its sensitivity to segregated impurities playing the role of either embrittlers or cohesion enhancers [1,2,3]. These issues have far-reaching practical implications manifested in the mechanical and magnetic properties. As an example, let us mention the strengthening/embrittling energy of segregated sp-elements from the 3rd, 4th and 5th period at the Σ5(210) grain boundary in ferromagnetic *fcc* nickel and cobalt [2,3,4,5,6,7,8]. Similar topics are also studied experimentally when, for example, the diffusion and segregation of silver in copper Σ5(310) grain boundary were investigated [9] or the influence of boron (segregated at the GB) on fracture resistance of Ni_3_Al [10] was analyzed. For some impurities segregated in Ni, e.g. for S, semiempirical interatomic potentials have been constructed [11]. Recent trends and open problems in grain boundary segregation are discussed in the reviews [3,12].

In nickel-based alloys, aluminum is added to improve high-temperature strength and precipitation hardening [13]. For example, an increased concentration of aluminum in the 718Plus alloy enhances its tensile strength, but has a negative impact on its ductility [14]. The experimental results also reveal that the content of aluminum has a significant effect on the solvus temperature in nickel-based alloys. In IN738 superalloy, silicon segregates mainly in inter-dendritic regions and promotes the segregation of other elements [15].

In this work, we present an ab initio study of the tilt Σ5(210) grain boundary in ferromagnetic *fcc* nickel both in the clean state and with segregated Al and Si impurities accompanied by vacancies. We look for the preferred positions of impurities at the GB and analyze their effect on atomic arrangement and magnetism. Though the physical mechanisms behind GB embrittlement and strengthening have been studied in detail for some materials [16,17,18,19], the specific interactions between GBs, impurities and vacancies are usually not taken into account. In our approach to segregation, not only the final equilibrium state of the studied system is characterized, but we also explore the way how it was achieved. We provide a detailed discussion of two methods of equilibration of structural arrangement and show that some of the initial configurations may end up in equilibrium state far from the initial structure (migration and vanishing of vacancies).

## 2. Materials and Methods

**Figure 1:**
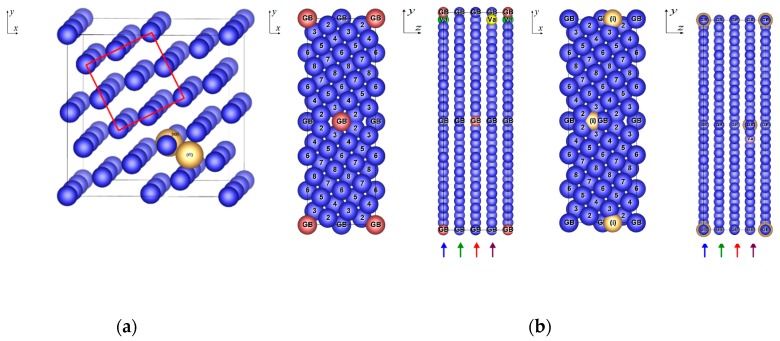
Supercells employed in the present calculations: (**a**) The *fcc* Ni_60_ supercell (Ni_60_–3 CSL cells behind each other) in a typical (3 CSL cells) arrangement having the lattice parameters √5*a*, √5*a*, 3*a*. The orange spheres denoted as iT (iO) occupy the tetrahedral (octahedral) positions of interstitial impurity atoms. The red square shows the position of the conventional *fcc* cell with 4 atoms. (**b**) The Σ5(210) grain boundary in *fcc* Ni supercell (GB Ni_120_, consisting of 6 CSL cells, 1×3×2 next to each other) having the lattice parameters √5*a*, 3√5*a*, 2*a*. The blue spheres correspond to Ni atoms, the spheres labelled by ‘‘GB’’ denote the GB plane, the red spheres occupy the position of substitutional impurity atoms, the orange spheres denoted as (i) mark the positions of interstitial impurity atoms. The green, yellow and violet spheres with label Va show the positions of vacancies in the structure with the substitutional Al^(s)^, substitutional Si^(s)^ and interstitial Si^(i)^ impurity, respectively. The numbers on atoms mark the layers counted from the GB plane. The planes with *z* = 0 and *z* = 0.5 (*z* = 0.25 and *z* = 0.75) are denoted by blue and red (green and violet) arrows.

To investigate the influence of the impurities, vacancies and grain boundaries on properties of ferromagnetic *fcc* nickel, the characteristics of bulk material were investigated first. In case of the bulk material (both without and with impurities), the *fcc* supercells with 60 (Figure 1a) or 120 atoms were used. The basic planes of the *fcc* supercells are constituted by (210) planes of standard *fcc* unit cell with 4 Ni atoms. Hence, *a* and *b* lattice parameters of the *fcc* supercells are √5*a* and the *c* lattice parameter is equal to an integer multiple of *a* (3*a* for Ni_60_ and 6*a* for Ni_120_), where *a* stands for the lattice parameter of the *fcc* Ni unit cell with 4 atoms. The cell with lattice parameters √5*a,* √5*a*, *a* is called a Coincident Site Lattice (CSL) cell. To get the *fcc* supercells with reasonable size of 60 or 120 atoms, the size of the CSL cell was increased 3 or 6 times in the direction of the *c* lattice parameter. Thus, the dimensions of the *fcc* supercell are √5*a*, √5*a*, 3*a* for a cell of the bulk material with 60 atoms (consisting of 3 CSL cells, Figure 1a) and √5*a*, √5*a*, 6*a* for a later employed cell of the bulk material with 120 atoms (consisting of 6 CSL cells).

The interstitial impurities were studied with respect to both octahedral (iO) and tetrahedral (iT) positions. In general, an atom in the octahedral position in the *fcc* unit cell is situated in octahedral site between six Ni atoms. One of four octahedral positions in *fcc* unit cell with 4 atoms, where our impurity was placed, is positioned between six Ni atoms at the centers of faces of the *fcc* cell. The fractional coordinates of such a position are *x* = *y* = *z* = ½. An impurity atom in the tetrahedral position in the *fcc* unit cell is situated in the tetrahedral site between four atoms. There are eight tetrahedral positions in the *fcc* unit cell with 4 atoms. One of these positions exhibits the fractional coordinates *x* = *y* = *z* = ¼. Supposing the touching spheres, the ratio between the radius of interstitial position (*r*) and the radius of the constituent atoms forming the *fcc* lattice (*R*) is *r*^iO^/*R =* 0.414 for iO position and *r*^iT^/*R =* 0.225 for iT position. It means that octahedral site is much larger than the tetrahedral one in *fcc* metals.

Further, the tilt Σ5(210) grain boundary in both a clean state and with impurities and vacancies (Figure 1b) was analyzed. This grain boundary was created by means of the rotation of two standard *fcc* cells with 4 atoms around the [001] axis by 53°. The method used for the construction of this supercell is called the Coincident Site Lattice principle [20]. In case of the supercells with GB, the supercell with 60 atoms (consisting of 3 CSL cells) has the lattice parameters √5*a*, 3√5*a*, *a*, and the supercell with 120 atoms (consisting of 6 CSL cells) has the lattice parameters √5*a*, 3√5*a*, 2*a* (Figure 1b). Because of the periodicity reasons, our GB supercells contain two grain boundaries oriented in the opposite direction, which is obvious from Figure 1b.

The above-mentioned structure is called B’.B’ [21] and is one of the known Σ5(210)[001] GB configurations. The other one (differing in the shape of interstitial sites) is the B.B structure. For this study, the B’.B’ configuration was chosen because it is supposed to have a lower GB energy, as deduced from atomistic studies [22,23].

All our calculations were performed at 0 K temperature within the Density Functional Theory (DFT) using the Vienna Ab initio Simulation Package (VASP) code [24,25,26] with Projector-Augmented-Wave – Perdew–Burke–Ernzerhof (PAW–PBE) potentials [27,28,29], i.e., in the Generalized Gradient Approximation (GGA). The optimum setting of computational parameters found by test calculations was as follows: the ENCUT parameter (the cut off energy defining the number of plane waves in the basis set) was 500 eV and the KSPACING parameter (which defines the number of k-points in the irreducible part of the Brillouin zone) was 0.1 Å^−1^. If not mentioned otherwise, the structure relaxations were performed as follows. The total energy was minimized with respect to the lattice parameters and atomic positions. At first, the conjugate–gradient method (using the Hellmann–Feynman forces acting on the atoms) and then the quasi–Newton method was employed to reach the required force limit. The Brillouin zone was sampled by the Monkhorst–Pack scheme and its integration was performed by Methfessel–Paxton or by the tetrahedron method, depending on whether relaxation or static calculation was carried out.

## 3. Results

### 3.1. Bulk Material

#### 3.1.1. Elemental Bulk Material

As the ferromagnetic *fcc* structure of elemental Ni is taken as the reference state in this study, its equilibrium properties (lattice parameters, atomic positions and total energy) were determined at first with the help of a sixty-atom supercell (Figure 1a). Similar analyses were performed for nonmagnetic *fcc* Al (4 atoms per cell) and nonmagnetic Si in diamond structure (8 atoms per cell) for the same reason. These three structures are taken here as the Standard Element Reference state (SER). The results obtained are summarized in Table 1.

Comparing the lattice parameters of the Ni_60_ structure with experimental data published in References [30,31], only a very small deviation within 0.6% and 0.1%, respectively, was found. The magnetic moment differs by 5.6%.

#### 3.1.2. Bulk Material with Impurities

The supercells including both substitutional and interstitial impurities were investigated using the following configurations: **Ni_59_Al^(s)^** (a structure with 59 atoms of Ni and one atom of Al in a substitutional position), **Ni_59_Si^(s)^** (a structure with 59 atoms of Ni and one atom of Si in a substitutional position) and **Ni_60_Si^(i)^** (a structure with 60 atoms of Ni and one atom of Si in an interstitial tetrahedral and octahedral position). The study of the configuration with the interstitial Al atom (Ni_60_Al^(i)^) was omitted as this structure is known as very unstable [35]. For comparison, we also studied the structure of **Ni_120_Si^(i)^** (a structure with 120 atoms of Ni and one atom of Si in a tetrahedral and octahedral interstitial position). The results of calculations of four *fcc* Ni supercells with interstitial Si (Ni_60_Si^(iT)^, Ni_120_Si^(iT)^, Ni_60_Si^(iO)^, Ni_120_Si^(iO)^) provide almost the same results: lattice parameters *a*, *b* and *c* differ by 0.57% at most and the volume per atom by 0.64%. The parameter *c* is two times larger in Ni_120_Si^(i)^ than in Ni_60_Si^(i)^, which is given by the increase of the number of atoms in the supercell.

The equilibrium lattice parameters and the total energies of all configurations were determined using the full relaxation of structure parameters. At first, the conjugate-gradient method and then the quasi-Newton method was used. The results obtained are summarized in Table 1.

The equilibrium lattice parameters and volume per atom (Table 1) show that the interstitial Si atom causes the largest volume increase in comparison with Ni_60_. Concerning the structures with substitutional Al and Si impurities, Ni_59_Al^(s)^ and Ni_59_Si^(s)^, the increase in volume per atom is larger for Ni_59_Al^(s)^. This can be explained by the fact that the atomic radius of the Al impurity is larger than that of the Si impurity.

Subsequently, the energies of formation of particular structures were calculated according to Equation (1):

(1) $$E^{f}=\left(E_{A_{x}B_{y}}-\left(x\frac{E_{A}}{N_{A}}+y\frac{E_{B}}{N_{B}}\right)\right)/N_{A_{x}B_{y}},$$

where $E_{A_{x}B_{y}}$ corresponds to the total energy of studied configuration, *x* denotes the number of atoms A and *y* denotes the number of atoms B. $E_{A}/N_{A}$ is the ground state energy per one atom A and $E_{B}/N_{B}$ is the ground state energy per one atom B. $N_{A_{x}B_{y}}$ is the total number of atoms in the compound A_x_B_y_.

For the studied structures in equilibrium arrangement, the following decreasing trend in the energy of formation related to *fcc* Ni, *fcc* Al and Si in diamond structure was found: Ni_60_Si^(iO)^ > Ni_60_Si^(iT)^ > Ni_120_Si^(iT)^ > Ni_120_Si^(iO)^ > Ni_60_ > Ni_59_Al^(s)^ > Ni_59_Si^(s)^, where the formation energy per atom of Ni_59_Al^(s)^ and Ni_59_Si^(s)^ differs only by 2% and is negative while the energies of formation of structures with interstitial Si are positive. This trend in energies of formation was confirmed by the calculations of the total energy of smaller cells, where the formation energies decrease in the sequence Ni_4_Si^(iT)^ > Ni_3_Al^(s)^ > Ni_4_ > Ni_3_Si^(s)^. However, these structures are not used for the study of impurities in this work as the concentration of impurities is too high in comparison with real materials. Based on the above-mentioned results, one can expect that both Si and Al would prefer the substitutional positions in bulk *fcc* Ni, which is in agreement with the results of a previous study [35].

Significant structural changes occurred during the relaxation of structures Ni_60_Si^(iT)^ and Ni_60_Si^(iO)^. The change in a position of atoms is the largest for structure Ni_60_Si^(iT)^. Here, the interstitial Si atom moves from the position between two planes towards one plane and changes the position of surrounding Ni atoms. After the relaxation, the new position of impurity cannot be considered as tetrahedral. The fractional coordinates of impurity in the 61 atomic unit cell (Figure 1a) has changed from *x =* 0.25, *y =* 0.25, *z =* 0.08 determining the tetrahedral position to nonspecific *x =* 0.23, *y* = 0.13, *z* = 0.17. The same trend can be observed for the structure Ni_60_Si^(iO)^. Here, the silicon impurity atom moves from the plane of atoms to the interplanar space and affects the surrounding Ni atoms and the relaxed position of the impurity atom is not octahedral. The fractional coordinates of this impurity in the 61 atomic unit cell (Figure 1a) has changed from *x* = 0.30, *y =* 0.40, *z* = 0.33 determining the octahedral position to nonspecific *x* = 0.40, *y* = 0.29, *z* = 0.38. The position of the Si impurity atom in structures Ni_120_Si^(iT)^ and Ni_120_Si^(iO)^ remains unchanged even after the relaxation. These structures can be considered as the structures with interstitial impurity atom occupying the tetrahedral or octahedral site. Comparing the larger structures with the smaller ones, it was concluded that the larger structures require smaller energies of formation (Table 1) which results in their larger stability. Comparing the structures Ni_120_Si^(iT)^ and Ni_120_Si^(iO)^, we can confirm the fact that the octahedral site is larger than the tetrahedral one, which results in greater stability of structure with the interstitial impurity in octahedral site where the smaller deformation of structure (displacement of adjacent Ni atoms) is needed to accommodate the impurity atom. The energy difference between Ni_120_Si^(iT)^ and Ni_120_Si^(iO)^ is 0.5886 kJ.mol^−1^, which predicts Ni_120_Si^(iO)^ as more stable.

Our results on energy of formation of Ni_59_Al^(s)^ and Ni_59_Si^(s)^ can be compared with the values obtained from References [33,34]. For this comparison, the values calculated as the linear combination of the literature data on the energies of formation of the cells with compositions Ni_3_Al, Ni_3_Si and pure Ni were employed. Hence, these results are not directly comparable with the data calculated for Ni_59_Al^(s)^ and Ni_59_Si^(s)^ structures. The energy of formation for Ni_59_Al^(s)^ calculated in this work and that obtained from the literature differ by 11.5%. In the case of Ni_59_Si^(s)^ structure, the difference between the calculated and reported energy of formation is 17.0%. In comparison with literature, our data show lower stability of studied structures.

It turns out that impurities significantly influence the magnetic moments of their neighboring Ni atoms (see Table 1) and they themselves obtain a small magnetic moment. The induced magnetic moment *μ* on the substitutional Al atom is slightly negative (*μ* = −0.018 μ_B_) with respect to Ni atoms and causes the decrease of magnetic moments of neighboring Ni atoms by 0.13 μ_B_. Substitutional Si (*μ* = −0.018 μ_B_) causes the decrease of magnetic moment of neighboring Ni atoms by 0.16 μ_B_ and interstitial Si in Ni_60_Si^(i)^ (*μ* = −0.021 μ_B_) induces the decrease of magnetic moment of neighboring Ni atoms by 0.35 μ_B_. In case of the Ni_120_Si^(i)^ (*μ* = −0.022 μ_B_), the decrease of magnetic moment of neighboring Ni atoms reaches 0.39 μ_B_. This significant impact of interstitial Si can be explained by the fact that in this case the neighboring atoms are much closer to the impurity than in the case of structures with substitutional impurity. Here, the largest decrease is caused by the interstitial atom of Si and the smallest decrease is due to the substitutional Al atom. Going further from the impurity, the magnetic moments of Ni atoms converge to the bulk value.

A similar study was also presented in References [2,32], where the change of magnetic moment of the 1st nearest neighbor (NN) nickel of Al^(s)^ (*μ* = −0.02 μ_B_) and Si^(s)^ (*μ* = −0.01 μ_B_) impurity are −0.10 μ_B_ and −0.14 μ_B_, respectively. These findings correlate very well with our values. Further, the lattice parameters *a*, *c* and the volume per atom found in the present and above-mentioned studies differ only by −0.45%, −0.64% and −1.54% (expressed in % of data found in this work) for Ni_59_Al^(s)^, respectively, and by −0.48%, −0.64% and −1.60% for Ni_59_Si^(s)^_._

#### 3.1.3. Bulk Material with Impurity and Vacancy

To study the effect of vacancies on the material properties, the basic quantities such as the vacancy-formation energy and the tendency to vacancy-impurity binding have to be evaluated. Hence, the relaxed configurations Ni_60_, Ni_59_Al^(s)^, Ni_59_Si^(s)^, Ni_60_Si^(iT/iO)^, and Ni_120_Si^(iT/iO)^ were relaxed again with one additional vacancy in their structure. The relaxation procedure was the same as for the systems without vacancy. The equilibrium lattice parameters, the volume per atom *V_at_*, the energies of formation of the structure *E^f^* and of the vacancy *E^f^(Va)* as well as the binding energies between impurity X and vacancy *E^b^(X;Va)* are summarized in Table 2. Subsequently, the effect of distance between the vacancy and impurity *D* on structural parameters, energetics and magnetic moments was analyzed.

Discussing the structure of configurations with and without vacancies, it is necessary to point out that all configurations of Ni_59_Si^(iT/iO)^+Va and Ni_119_Si^(iT/iO)^+Va structure (except for the fourth one of Ni_119_Si^(iT)^+Va and the second ones of Ni_119_Si^(iO)^+Va and Ni_59_Si^(iO)^+Va, all of them denoted by bold text in Table 2) are not stable and they transform to Ni_59_Si^(s)^ or Ni_119_Si^(s)^ structure. In such case, the vacancy is occupied by interstitial Si atom or Ni atom and disappears. In this way, the interstitial Si atom becomes substitutional and the structure achieves the properties comparable with the Ni_59_Si^(s)^ structure. In case of the third configuration of both structures, the values of *D* parameters differ. This comes from the fact that one of them is the starting value (denoted by *) and the other is the equilibrium one.

The lattice parameters almost do not depend on the position of the vacancy (not discussing the structures Ni_119_Si^(iT)^+Va where the equilibrium lattice angles are not 90°). They vary within one hundredth, exceptionally one tenth, of percent. The lattice parameters of the configurations, where the “recombination” has occurred, exhibit the differences amounting up to 1% from values calculated for structures without vacancy.

In structures with a stable vacancy, the vacancy causes a decrease of the cell volume but an increase of the volume per atom, which is obvious because the structure relaxation cannot cause the decrease of volume in such extent, which would correspond to the volume per atom of missing particles.

In case of Ni_58_Al^(s)^+Va and Ni_58_Si^(s)^+Va structure, the minimum-energy configurations are those with the vacancy and impurity being the nearest neighbors. In case of the structure Ni_59_Si^(iT)^+Va, the relaxation of all starting configurations results in the same final arrangement, namely Ni_59_Si^(s)^. In Ni_119_Si^(iT)^+Va, the only stable configuration with Va is that one with the distance between the vacancy and impurity being 5.2 Å, i.e. Si and Va are in the 4th nearest neighbor position. In Ni_59_Si^(iO)^+Va, the stable configuration is that one with the distance between the vacancy and impurity being 3.5 Å and in Ni_119_Si^(iO)^+Va being 3.1 Å, i.e. Si and Va are in the 2nd nearest-neighbor’s position. Analyzing the stability of vacancy in the vicinity of the impurity, it must be concluded that substitutional impurities prefer to be in the vicinity of the vacancy. On the other hand, the Si interstitial must be more distant from the vacancy to prevent the recombination of defects resulting in disappearance of vacancy and change of the impurity site from interstitial to substitutional (only three configurations were able to retain Va in their structure).

According to the values of the energies of formation provided in Table 2, it is straightforward that the interstitial Si atoms with a vacancy in their neighborhood prefer the recombination of defects resulting in a structure without a vacancy and with substitutional Si. If the vacancy is too distant from the interstitial Si atoms, the interstitial position of Si can be retained which results in the positive value of the energy of formation which are comparable for both types of the interstitial position in large cells.

The energies of vacancy formation of structures Ni_59_+Va, Ni_59_Al^(s)^+Va, Ni_59_Si^(s)^+Va, Ni_59_Si^(iT/iO)^+Va and Ni_119_Si^(iT/iO)^+Va were computed according to Equation (2):

(2) $$E^{f}\left(Va\right)=\left(E_{bulk+Va}+E_{1fccNi}\right)-E_{bulk}$$

and are listed in Table 2. It is obvious that the vacancy formation causes the structure destabilization characterized by positive values of *E^f^(Va)*. For the structures Ni_59_+Va, Ni_58_Al^(s)^+Va, and Ni_58_Si^(s)^+Va, the increase in the total energy due to the *E^f^(Va)* contributions amounts to about 1.4 eV.Va^-1^. In case of the structures with interstitial Si, the situation is more complicated. For the Ni_59_Si^(iT)^+Va and the first two configurations of Ni_119_Si^(iT)^+Va, a decrease in the formation energy of -3.05 eV.Va^-1^ and of about −4 eV.Va^−1^ was found. The third configuration (*D* = 4.5 Å) of Ni_119_Si^(iT)^ exhibits even the formation energy of −1.2 eV.Va^−1^, which can be caused by the change of structure during the relaxation where the equilibrium angles are *α* = 90.00°, *β* = 89.99° and *γ* = 92.43°. The formation energies of the structures with octahedral impurity (Ni_59_Si^(iO)^+Va and Ni_119_Si^(iO)^+Va), except the second configuration, are in the range from −3.2 to −4.8 eV.Va^−1^. However, the stabilization in all above-mentioned configurations with interstitial impurities is caused by the formation of the Ni_59_Si^(s)^ or Ni_119_Si^(s)^ structure rather than by the Va formation itself. In case of the fourth configuration of Ni_119_Si^(iT)^+Va and the second of Ni_59_Si^(iO)^+Va and Ni_119_Si^(iO)^+Va, the energy of vacancy formation reaches low positive values of 0.5, 1.1, and 1.4 eV.Va^−1^. This is natural because here the created empty (Va) space compensates the destabilization caused by the tension originating from the interstitial impurity. Simultaneously, the most stable configurations of all structures reveal the lowest values of *E^f^(Va)*.

The influence of the position of vacancy in a particular configuration on its energy of formation *E^f^* and on the vacancy-formation energy *E^f^(Va)* is very small except for the cases with interstitial Si. For the structures represented by the supercells Ni_58_Al^(s)^+Va and Ni_58_Si^(s)^+Va, the scatter in the energy of formation of vacancy is within several hundredths of eV.Va^−1^. This means that there is no special interaction between the substitutional impurity and vacancy in comparison with bulk Ni (this is confirmed by similar values of *E^b^(X;Va)* as discussed below). For all configurations of structures with interstitial impurity (except for the fourth one of Ni_119_Si^(iT)^+Va and second ones in Ni_59_Si^(iO)^+Va and Ni_119_Si^(iO)^+Va), the formation energy of Va cannot be evaluated (it corresponds to the energy of structure transformation).

Regarding the literature data, our results are comparable with experimental vacancy-formation energies of 1.7 eV [36], 1.78 eV and 1.79 eV and with other calculated data (see Reference [37] and references therein). Another experimental value of formation energy of vacancy in bulk Ni is 1.73 eV [38]. Theoretical values found in literature amount to 1.41 eV (VASP, GGA, Perdew–Wang) [39], 1.379 eV [40], 1.48 eV (QE GGA-PBE, mag.) [41], and 1.48 eV (GGA-PBE) and 1.63 eV (Local Density Approximation (LDA)) (both Reference [42]) followed by similar data of 1.57 eV [43], 1.58 eV [44], 1.31 eV [45], 1.6 eV [46], 1.279 eV [47], 1.77 eV [37] and 1.39 eV [35].

From the energetical point of view, the further quantity – the binding energy of two defects (impurity and vacancy) – can be evaluated by Equation (3):

(3) $$E^{b}\left(X;Va\right)=\left(E_{bulk+X+Va}+nE_{1fccNi}\right)-\left(E_{bulk+X}+E_{bulk+Va}\right),$$

where the first term describes the energy of material with interacting defects, the second term is the correction by means of the energy of bulk Ni and the third and fourth term correspond to the energy of noninteracting defects placed in different computational cells. The calculated values are given in Table 2.

In case of the most stable structures with substitutional impurities, the binding energy *E^b^(X;Va)* reaches the values of −0.0451 and −0.1110 eV.X^−1^Va^−1^. In case of interstitial Si, these values are much lower: −0.1578 eV.X^−1^Va^-1^ for Si in iT position and −0.2343 and 0.0012 eV.X^−1^Va^−1^ for Si in iO position. Nevertheless, the most negative values are obtained for the most stable configurations (1st NN for substitutional impurity, 3rd NN for iT impurity and 2nd for iO impurity). The negative values can be interpreted as the tendency to the binding between vacancy and impurity.

**Figure 2:**
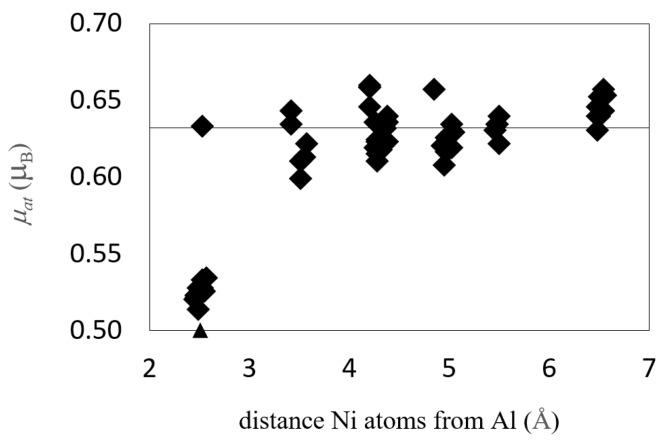
The dependence of the magnetic moment of Ni atom on its distance from Al^(s)^ in the structure Ni_58_Al^(s)^+Va (the vacancy is situated in the first nearest neighbor position of the Al atom (*D* = 2.4594 Å)). The line corresponds to the calculated value of magnetic moment of bulk *fcc* Ni 0.632 μ_B_.

It was found that the presence of a vacancy causes the increase of magnetic moments of neighboring Ni atoms by 0.01–0.03 μ_B_. The size of this effect depends on the mutual position and interplay of all three “particles”: Ni atom, impurity atom and vacancy. Usually the “magnetic-moment-decreasing” effect of antiferromagnetic impurity (which is induced by surrounding Ni atoms because originally the Si and Al impurities are nonmagnetic) is bigger than the “magnetic-moment-increasing” influence of vacancy (Figure 2). It was proved that the influence of impurities and vacancies on Ni magnetism gradually decreases with distance and the magnetic moment converges to bulk-material value. The bulk values were reached when the distance of a Ni atom from the impurity was equal to 3.5–5.0 Å.

There can be a difference in the magnetic moments of atoms with the same distance from the impurity atom, which is caused by the presence of vacancy in the structure (Figure 2). Here, the Ni atoms lying closer to the vacancy possess higher magnetic moments than those lying further from it but having the same distance from Al impurity.

### 3.2. Clean Grain Boundary

#### 3.2.1. Structure Arrangement

After the studies of bulk material, a supercell with Σ5(210) grain boundary including sixty atoms of nickel (GB-Ni_60_) (Figure 1b) and also the derivatives of the bigger cell (GB-Ni_120_) were studied. Because of actual scientific discussions, how to properly optimize structural parameters in materials with grain boundaries [48] detailed tests of two relaxation methods were performed to confirm the reliability of our further approach. These tests are significant as their conclusions are transferable to other systems. The first method (Method 1) determines the equilibrium lattice parameters and total energy from the automatic full relaxation of structure parameters based on the minimization of forces acting on particular atoms. The other one (Method 2) consists in the evaluation of a set of calculations where the two lattice parameters (in the plane of the GB *a* and *c*) are kept fixed, one parameter (dimension perpendicular to the boundary, *b*) is changed to various values and the simultaneous relaxation of atomic positions is performed for each setting of lattice parameters. In this case, the equilibrium structure and energy were determined with respect to a) minimum energy (Method 2a) or b) the zero stress along the dimension perpendicular to the boundary (Method 2b).

The full relaxation corresponds to a situation in small grains, where also the bulk region is affected by the grain boundary, which plays a major role. The situation in larger grains is different. Here, the bulk interiors keep their own bulk values (lattice constant) so that a restricted relaxation (when the dimension perpendicular to the GB is relaxed and the interface dimensions in the GB plane are kept equal to the bulk values) should provide a better description.

Both above mentioned relaxation approaches (Method 1, Method 2) were used for the GB-Ni_60_, GB-Ni_120_, GB-Ni_118_Al_2_^(s)^, GB-Ni_118_Si_2_^(s)^ and GB-Ni_120_Si_2_^(i)^ supercells. In case of the structures with an impurity, the large cells were used only to ensure the conservation of bulk-material properties between the grain boundaries. It was confirmed that the second method provides almost the same lattice parameters as the first one regardless of the method of determination of equilibrium structure. There is a difference of 0.7% between the equilibrium cell volumes obtained using the full relaxation and using the second approach for GB-Ni_60_. The *b*/*a* ratio and the free volume also do not change very much (see Table 3 in next chapter). Because of these insignificant differences, the both relaxation methods can be considered as equivalent and the Method 1 was used in the subsequent calculations.

**Figure 3:**
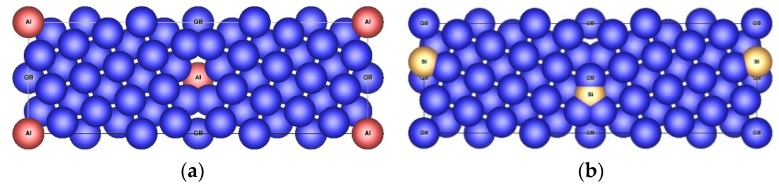
Equilibrium GB supercells containing impurities: (**a**) GB-Ni_118_Al_2_^(s)^, (**b**) GB-Ni_120_Si_2_^(i)^. The blue spheres correspond to Ni atoms, the spheres labelled by ‘‘GB’’ denote the GB plane, the red spheres occupy the position of substitutional impurity atoms, the orange spheres denoted as Si mark the positions of interstitial impurity atoms.

For better orientation in the following text, we will use the word “layer” for layers of the (210) type (those parallel with the GBs) and the word “plane” for planes of the (001) type (those perpendicular to GBs and lying in the plane of the paper in Figure 3, i.e. those with GB image).

**Figure 4:**
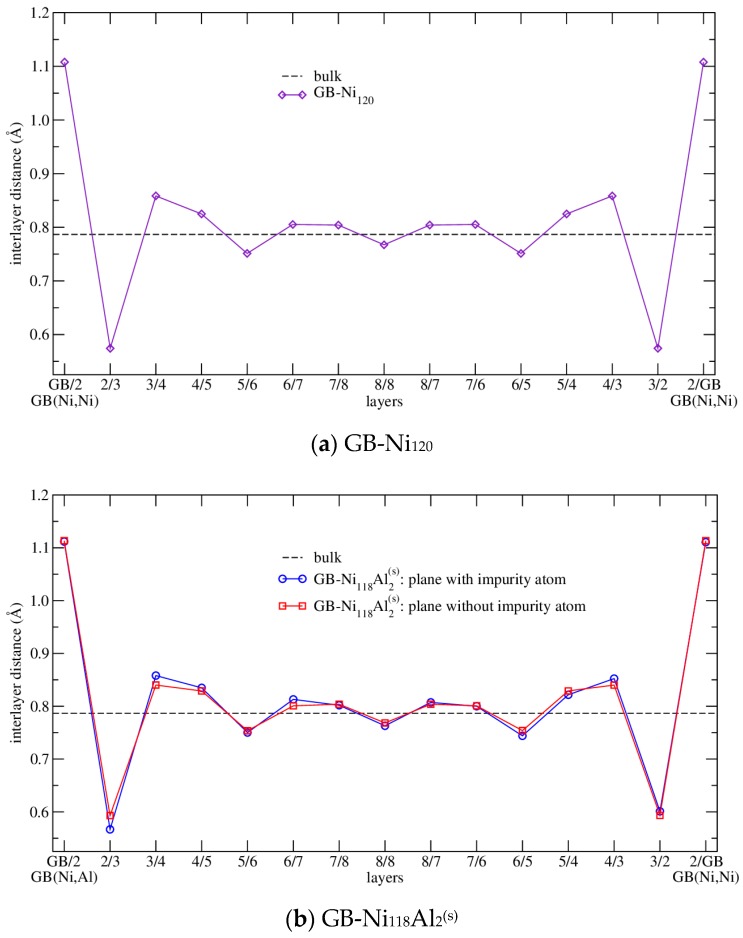

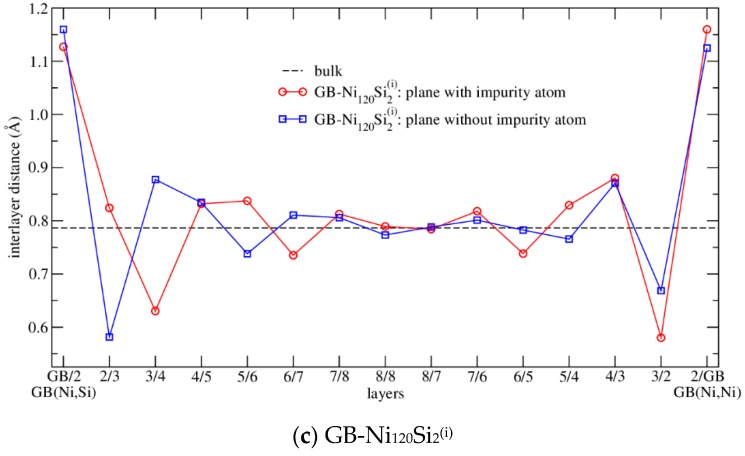
The interlayer distances in supercells with a GB: (**a**) GB-Ni_120_, (**b**) GB-Ni_118_Al_2_^(s)^, (**c**) GB-Ni_120_Si_2_^(i)^. The violet line with diamonds corresponds to a clean GB in elemental *fcc* Ni. The blue (red) line with squares (circles) characterizes the planes without (with Al or Si) impurity, which are parallel to the plane of paper in structures shown in Figure 3. The exact meaning of expression plane is defined in the text. GB(X,Ni) denotes the GB with impurity X (X = Al^(s)^, Si^(i)^) and GB(Ni,Ni) the clean GB. The lines do not reflect any physical meaning; they are used to guide the eye only. The dashed line corresponds to the interlayer distance in ferromagnetic *fcc* Ni (this work). For a better demonstration of results in a case of (b) and (c), the plane with impurity atom is always presented so that there is an impurity on the left part of the figures. This was achieved by the shift ½ of the lattice parameter *b* of lines representing the results of some planes with impurities (the middle plane of GB-Ni_118_Al_2_^(s)^ or the second plane of GB-Ni_120_Si_2_^(i)^) and one without impurity (the middle plane of GB-Ni_120_Si_2_^(i)^) see Figure 1. This figure does not contain the system GB-Ni_118_Si_2_^(s)^ as it provides results similar to data presented for system GB-Ni_118_Al_2_^(s)^ in part (b).

The dependence of interlayer distances in a supercell containing a clean GB with respect to the type of atomic layers is displayed in Figure 4a (next section) and numerical data are provided in Table S1 in the Supplemental Material. The first interlayer distance GB/2 (the distance between the grain boundary and the second atomic layer) expanded to 1.10 Å and is by about 40% larger than the calculated bulk value of 0.79 Å. On the other hand, the second interlayer distance 2/3 (the distance between the second and the third atomic layer) contracted to 0.57 Å and is by about 27% smaller than the value for the bulk *fcc* nickel. The interlayer distance 3/4 is expanded again, namely to 0.86 Å, i.e. it is about 9% larger than the bulk value, and so on. From the sixth layer on, the interlayer distances closely oscillate around the calculated bulk value. This proves that the supercell used in the calculations is large enough to avoid the interactions between the present two grain boundaries. Our results are also in agreement with the data calculated by Všianská and Šob [2].

#### 3.2.2. Energetics

The GB energy (energy of formation of a GB) *γ_GB_* is defined as the energy needed to create 1 m^2^ of GB in the bulk material. Within the ab initio approach, it can be calculated as the difference of the total energies of two supercells: one with the grain boundary (*E_GB_*) and the other one without it (*E_bulk_*). This difference is then divided by the doubled area of the GB in the cell *S* (because there are two boundaries per cell in the periodic system) [49]. This results in the following Equation (4):

(4) $$\gamma_{GB}=\frac{E_{GB}-E_{bulk}}{2S}.$$

The GB energy *γ_GB_* (see Table 3 in the next chapter) calculated with the help of clean GB-Ni_60_ and GB-Ni_120_ supercell is in a very good agreement with data obtained from literature [2,50]. The energy per atom of Ni in the supercell with grain boundary is higher by 1.34% than the energy per atom in bulk Ni for both GB-Ni_60_ and GB-Ni_120_, which means that the grain boundary is unstable with respect to the bulk material.

#### 3.2.3. Magnetism

**Figure 5:**
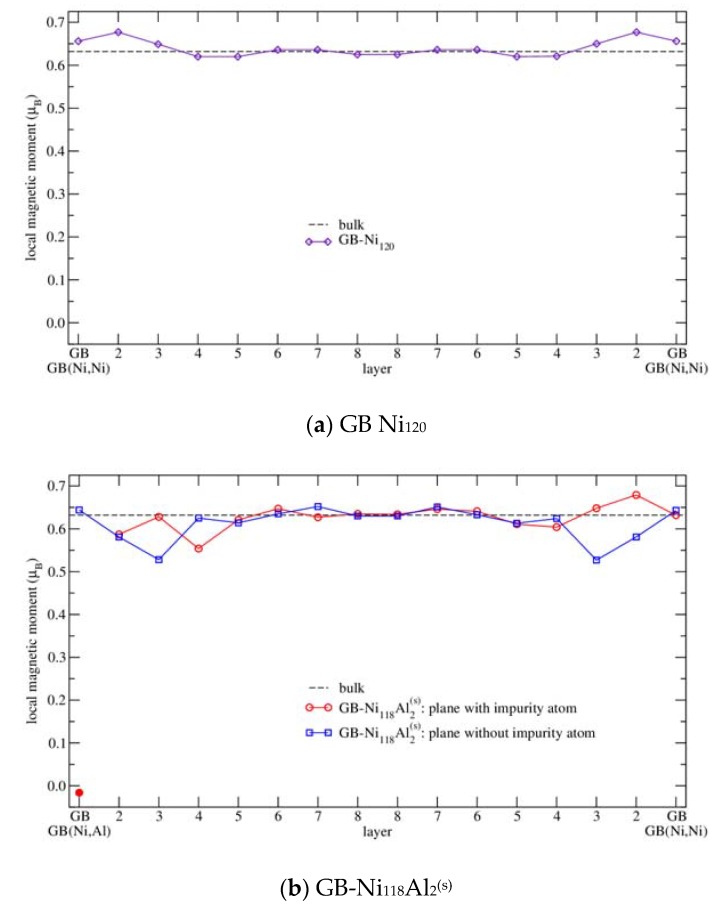

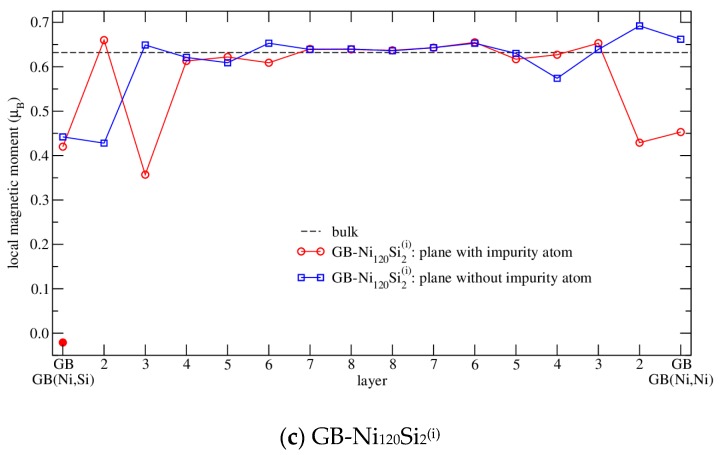
The dependence of magnetic moment of Ni atom on the number of the atomic layer: (**a**) GB-Ni_120_, (**b**) GB-Ni_118_Al_2_^(s)^, (**c**) GB-Ni_120_Si_2_^(i)^. The violet line with diamonds corresponds to the Σ5(210) GB in elemental *fcc* Ni. The blue (red) line with squares (circles) corresponds to the planes without (with Al or Si) impurity, which are parallel to the plane of paper in structures shown in Figure 3. The exact meaning of expression plane is defined in the text above. GB(X,Ni) denotes the GB with impurity X (X = Al^(s)^, Si^(i)^) and GB(Ni,Ni) the clean GB. The lines do not reflect any physical meaning; they are used to guide the eye only. The dashed line shows the magnetic moment of *fcc* bulk Ni of 0.632 μ_B_ calculated in this work. The full red circle corresponds to the magnetic moment of Al (Si) impurity. This figure does not contain the system GB-Ni_118_Si_2_^(s)^ as it provides results similar to data presented for system GB-Ni_118_Al_2_^(s)^ in part (**b**). For a better demonstration of results in case of (**b**) and (**c**), the plane with impurity atom is always presented so that there is an impurity on the left part of the figures. This was achieved by the shift of lines representing the results of some planes with impurities (the middle plane of GB-Ni_118_Al_2_^(s)^ or the second plane of GB-Ni_120_Si_2_^(i)^) and one without impurity (the middle plane of GB-Ni_120_Si_2_^(i)^) by ½ of the lattice parameter *b*.

Concerning the magnetism, it was found that the magnetic moment of Ni is strongly influenced by the presence of the grain boundary as it increases up to its maximum 0.677 μ_B_ in the layer next to the GB in case of GB-Ni_60_ supercell. This value is by 7% higher than the bulk calculated value of 0.632 μ_B_, which is in agreement with the enhancement found in [2]. Then, the magnetic moments of Ni atoms located farther from the grain boundary gradually decrease to the bulk value (Figure 5a in the next chapter).

In case of GB-Ni_120_ supercell (Figure 5a), the magnetic moment of Ni atoms situated at the grain boundary (0.656 μ_B_) is by 3.7% higher than the calculated magnetic moment of Ni atom in the bulk Ni (0.632 μ_B_). And again, the atoms in the layer 2 exhibit a larger enhancement of magnetic moment that is higher by 7.1% in comparison with the bulk value. Atoms in the 3rd layer have magnetic moment higher by 2.8%. However, the Ni atoms in the 4th layer exhibit a value by 2.0% lower than the magnetic moment of Ni atoms in the bulk. Then, the values of the magnetic moments of atoms located in further layers decrease and oscillate around the bulk value of 0.632 μ_B_. These values are also in a very good agreement with those published in Reference [2].

### 3.3. Grain Boundary with Impurities

#### 3.3.1. Structure Arrangement

To find the most preferable positions of impurities in Ni material, the properties of the supercells with Σ5(210) grain boundary with two impurity atoms were investigated. For this purpose, three different configurations of grain boundary were proposed: GB-Ni_118_Al_2_^(s)^ (with two substitutional Al atoms), GB-Ni_118_Si_2_^(s)^ (with two substitutional Si atoms) and GB-Ni_120_Si_2_^(i)^ (with two interstitial Si atoms in position at GB layer visualized in Figure 1b). The equilibrium structure data are provided in Table 3 and the corresponding structures are displayed in Figure 3.

Comparing the values of *V_at_* for various GB configurations, it is obvious that they are comparable, which means that the introduction of impurity does not influence significantly the size of the structure. The lowest value was obtained for the structure with interstitial impurities. It may be seen that there is an increase of volume related to the GB introduction. This effect can be quantified by means of the excess free volume:

(5) $$V^{f}=\left(V_{GB}-V_{bulk}\right)/2S,$$

which is a GB specific property [51,52] like the GB energy discussed below. The value of *V^f^* for clean GB obtained from automatic relaxation is 0.26 Å^3^.Å^−2^ (see Table 3). This value is identical with the values obtained by the same method for structures with substitutional impurities. This means that the structures need some additional volume to form the GB. The value of *V^f^* for structure with interstitial impurity is 0.11 Å^3^.Å^−2^ and it is much lower than the values corresponding to other structures. This means that the big change in volume of bulk structure caused by introduction of the interstitial impurity (see Table 1) is consequently also used for the formation of GB. In case of the GB-Ni_60_ supercell, the values of *V^f^* (Table 3) are fully comparable with the values of other structures. This is caused by the fact that both the *V_G_*_B_-*V*_bulk_ and 2*S* for GB-Ni_120_ modifications are two times larger than for GB-Ni_60_. The comparability between the results of Method 1 and 2 is not ideal, as the two lattice parameters (in the plane of the GB) and hence also the value of 2*S* are kept fixed in Method 2.

The first coordination sphere of the substitutional impurity is very similar to that of atoms in elemental bulk *fcc* Ni. Nevertheless, the interatomic distances are scattered a little bit and increased with respect to ideal value (2.475 Å). In case of Al^(s)^, they reach values in the range from 2.585 to 2.640 Å. The first coordination sphere of substitutional Si^(s)^ is even more scattered and slightly enlarged (2.504–2.645 Å). On the other hand, in the structure with interstitial Si, several Ni atoms in the neighborhood of Si atom changed their positions to make more space for the impurity. The coordination sphere is slightly compressed, and the scatter exhibits the largest range here (2.177-2.444 Å) here. The Ni atoms in the third layer of GB with the same *z* coordinate as Si atom are most affected. The equilibrium position of these Ni atoms differs from the initial position by 0.012% in *y* direction. This movement causes the increase of the distance between Ni and Si atom.

Analyzing the interlayer distances (see Figure 4 and Table S1 in Supplemental Material), it is obvious that they are comparable with the results presented in the papers [2,49], i.e. there is a big increase in the distance between the GB and layer 2 (GB(X,Ni)/2, GB(Ni,Ni)/2) (with a maximum for the GB-Ni_120_Si_2_^(i)^ structure) and a significant decrease in the distance between the layers 2 and 3 (2/3) in all studied configurations. The distances between next layers are not so much affected by the presence of the impurity and they converge with small oscillations to the bulk value. As there are two types of planes (without and with impurity) in the supercell, there are also two types of lines (blue with squares and red with circles) depicted in Figure 4. In case of the substitutional impurities (Figure 4b), these curves mostly overlap. The red one (plane without impurities) is symmetric with respect to the center of the picture. The blue curve (plane with impurities) reveals some deviations from this symmetry, which reflects the fact that the GBs are not fully equivalent. However, as the deviations are very small, it can be concluded that the type of GB (clean or with impurity) has a negligible impact on the interlayer distances. These conclusions are valid also for the system GB-Ni_118_Si_2_^(s)^ which is not displayed in Figure 4. In case of the interstitial Si (Figure 4c), the figure looks different. This is caused by the fact that the two impurity atoms are positioned in the supercell asymmetrically (see Figure 1b). The most obvious difference may be seen in the case of the blue line in the left part of the figure, which reveals a strong asymmetry. This reflects the fact that the interstitial impurities influence the structure much stronger than the substitutional ones.

Again, our findings are in a good agreement with the data calculated by Všianská and Šob [2] and we recalculated them to have a benchmark. Nevertheless, the present results are not fully comparable with those in [2] as the concentrations of the impurities in the layers are not the same. The ratio of the number of atoms Ni:X^(s)^ (Ni:X^(i)^) in one layer is 3:1 (4:1) in our work and 0:2 (2:2) in Reference [2], which means that our supercells exhibit a considerably lower concentrations of impurities which is closer to real systems. In the case of structure with a substitutional impurity in Reference [2], there are 56 atoms of Ni and 4 atoms of impurity. The impurity atoms are forming the monolayer at the GB layer including 2 impurity atoms at each GB. For the structure with an interstitial impurity in Reference [2], there are 60 atoms of Ni and 4 atoms of impurity. Each GB is occupied by 2 atoms of Ni and 2 atoms of impurity. We also have to take into account that the GB cells used by Všianská and Šob [2] are usually smaller than the cells presented in this work.

The differences may be characterized as follows. In comparison of the interlayer distances of structures with GB and impurity calculated by Všianská and Šob [2] and presented in this work, it can be said that the interlayer distances calculated for structures GB-Ni_120_ and GB-Ni_118_Al_2_^(s)^ differ in maximum by 2%. For the structure GB-Ni_118_Si_2_^(s)^ the 2/3 interlayer distance is by 26% larger and 3/4 interlayer distance is by 12% smaller than the same interlayer distances in the paper [2]. Additionally, for the structure GB-Ni_120_Si_2_^(i)^, there are significant differences in GB/2 interlayer distance and 2/3 interlayer distance. Both values are smaller than data published in [2]; the difference is 10% and 15%, respectively.

#### 3.3.2. Energetics

Based on the total energies, we can estimate the preference of Si to occupy the substitutional or interstitial positions at the studied GB using the following equation:

(6) $$\Delta E=\frac{\left(E_{GB-Ni_{118}Si_{2}^{\left(s\right)}}+2E_{1fccNi}\right)-E_{GB-Ni_{120}Si_{2}^{\left(i\right)}}}{2},$$

where the negative value of the Δ*E* corresponds to the preference of the substitutional position. This is not the case of Si impurity at the GB, where Δ*E* = 0.4798 eV.atom^−1^ of Si. Hence, in case of the Si the occupational preference is changed to interstitial positions. The fraction of Si atoms located in substitutional positions at the Σ5(210) GB will be therefore negligible.

The energy per one atom in the supercell with a grain boundary including the impurity atoms is higher by 0.3–1.4% than the energy of bulk structure with impurities. The energy difference (*E_GB_*-*E_bulk_*) is lowest in case of the GB-Ni_120_Si_2_^(i)^ supercell and the largest in GB-Ni_118_Si_2_^(s)^.

Furthermore, we can also analyze the GB energies *γ_GB_* (Equation (4), Table 3). Here, the relaxation methods 1, 2a and 2b give almost the same results for structures with impurities, which differ within 2.2% for Ni_120_Si_2_^(i)^. In a case of structures without impurity the GB energies given by the three types of relaxation differs less. It is 1.2% for GB-Ni_60_ and even 1.0% for GB-Ni_120_. Because the differences are not significant, this is a further argument supporting the statement that all methods of relaxation used here are equivalent. In case of the GB-Ni_60_ and GB-Ni_120_ supercell, the values of *γ_GB_* (Table 3) are fully comparable because both the *V_GB_*-*V_bulk_* and 2*S* for GB-Ni_120_ are two times larger than for GB-Ni_60_. According to the presented values of GB energy, it can be said that the energy related to the formation of GB is increasing in this sequence: GB-Ni_120_Si_2_^(i)^ < GB-Ni_60_ = GB-Ni_120_ < GB-Ni_118_Al_2_^(s)^ < GB-Ni_118_Si_2_^(s)^, which means that the formation of the GB is easiest in the structure with interstitial Si, even easier than in elemental *fcc* nickel, and that substitutional impurities make the GB formation most difficult. The *γ_GB_* for GB-Ni_120_Si_2_^(i)^ configuration is very small, which supports the stability of the structure with interstitial Si with respect to the structure containing substitutional Si. Our calculated GB energy *γ_GB_* in elemental Ni (1.29 J.m^−2^, Table 3) agrees very well with the value of 1.23 J.m^−2^ published in Reference [2], which was obtained by the VASP code within the same GGA approach. We also obtained a reasonable agreement with a value of 1.43 J.m^−2^ reported in Reference [50], where the WIEN2k code with GGA approach in the PBE96 parametrization was employed, the $\sqrt{5}a$, 2$\sqrt{5}a$, *a* cell was used but the spin polarization was omitted. The GB energy was also studied by atomistic simulations [53] providing the values of 1.23 J.m^−2^ and 1.28 J.m^−2^. These values also compare well with our data. To the best of our knowledge, no experimental value for GB energy of the Σ5(210) GB in Ni is available, and hence a comparison of theoretical calculations with experiment is not possible yet.

The other energetic quantity, which can characterize the properties of the GB and/or impurities is the segregation energy (the binding energy between impurity and GB). The segregation energy of an impurity at a GB can be measured [3] and is defined as the lowering of the energy of the system when solute atoms go from the bulk material to the GB. It can be expressed by the Equation (7):

(7) $$E^{seg}\left(X\right)=\frac{\left(E_{GB+X}-E_{GB}\right)}{2}-\left(E_{bulk+X}-E_{bulk}\right)=E_{XatGB}-E_{Xinbulk},$$

where $E_{GB+X}$ and $E_{GB}$ are the energies of the GB structure with and without impurity atoms (both containing the same number of Ni atoms) and $E_{bulk+X}$ and $E_{bulk}$ are the total energies of a bulk material with and without impurity (both containing the same number of Ni atoms). The energy difference for bulk material has to be subtracted here to eliminate the energetics of adding impurity to the bulk material [49,54]. $E_{XatGB}$ and $E_{Xinbulk}$ are the total energies of one atom of impurity at GB and in a bulk material. The results are summarized in Table 4.

The segregation energies of substitutional Al^(s)^ atom and interstitial Si^(i)^ atom are negative for all methods of relaxation, which means that both impurities prefer a location in the neighborhood of GB to that in the bulk material. These values also support the stability of these configurations. The segregation energy of substitutional Si^(s)^ atom is positive, so that substitutional silicon prefers to stay in the bulk. However, this arrangement is less stable than the structure with interstitial Si^(i)^ as stated above. Equation similar to Equation (7) was also used by Yamaguchi et al. in Reference [55].

Unfortunately, there are no experimental data available in literature regarding the segregation enthalpies of the Σ5(210) GB for Si and Al. The only data found are those for Al at specific GB types where the values −2, −4 and −5 kJ.mol^−1^ were obtained for the surface (001), (123) and (015), respectively (see Reference [3] and references therein). Thus, our calculated segregation energies are 6.9, 3.5 and 2.8 times higher than those obtained from experiments. It should be mentioned that these values correspond to different types of GBs and therefore our comparison of calculations and experiment is somewhat limited. It should also be considered that other configurations of the Σ5(210) GB with Si and Al atoms may occur in reality and in the present work we examined only one of them.

The obtained results for the segregation of Al^(s)^ and Si^(i)^ at the Σ5(210) GB can be also compared with other computational studies [2,50]. The calculated values from Reference [2] and [50] correspond to ours very well as they differ only by hundredths and tenths of eV in case of Al^(s)^ and Si^(i)^, respectively.

The calculations in Reference [2] were performed using the same exchange-correlation energy but a smaller cell with a higher concentration of impurities at the GB (impurity monolayer). In reference [8] norm-conserving pseudopotentials and local density approximation for the exchange-correlation potentials were employed. The method used in Reference [50] is based on the full-potential linearized augmented plane wave method with the generalized gradient approximation of PBE96 parametrization [29] and, again, a smaller cell with even higher concentration of impurities than in [2] (impurity monolayer at GB) is applied. Moreover, in [50], the calculations were performed as nonmagnetic. Considering the above-mentioned differences in the approach in Reference [50] and configurations [2], the agreement is reasonable and confirms the behavior of segregating elements at the GB, i.e. Al as substitutional and Si as interstitial.

#### 3.3.3. Magnetism

**Figure 6:**
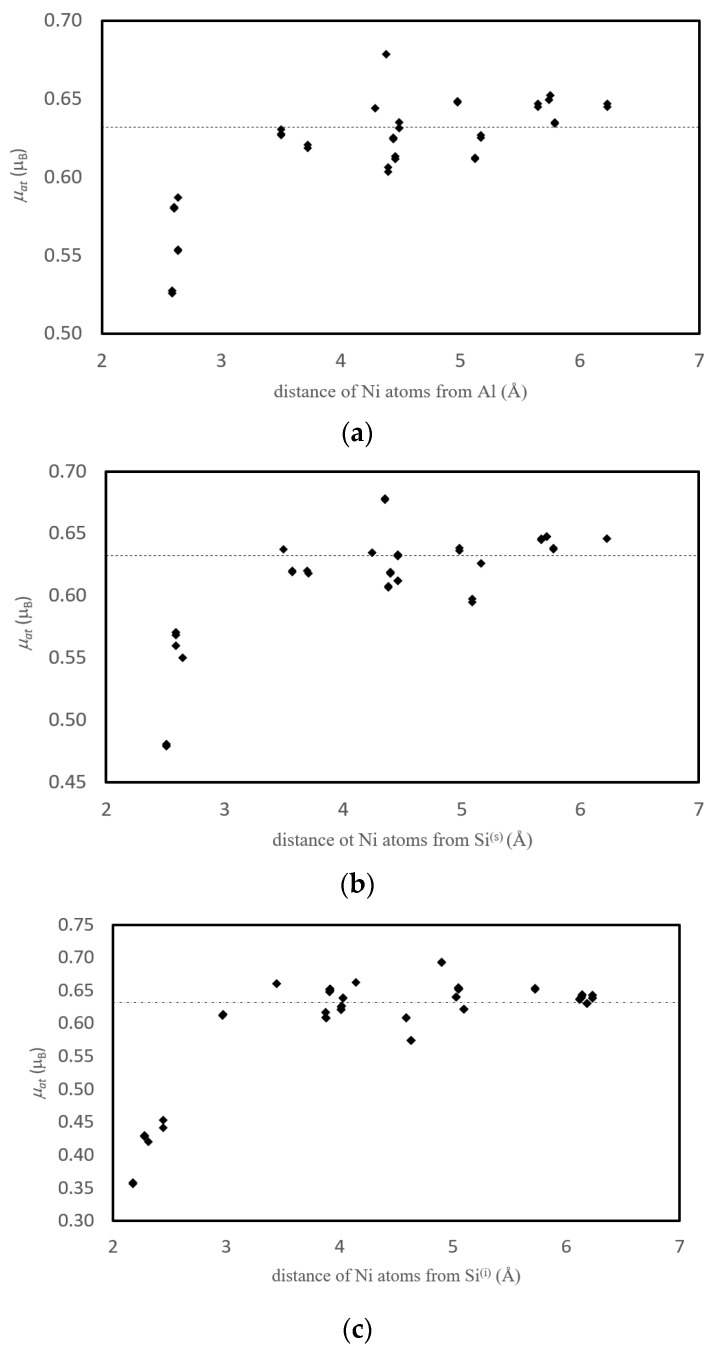
The dependence of magnetic moment of Ni atom on the distance from the impurity: (**a**) GB-Ni_118_Al_2_^(s)^, (**b**) GB-Ni_118_Si_2_^(s)^, (**c**) GB-Ni_120_Si_2_^(i)^. The dashed line corresponds to the magnetic moment of elemental *fcc* bulk Ni of 0.632 μ_B_ calculated in this work.

As it was shown in previous chapters, the presence of impurities and grain boundaries has a very strong impact on the value of magnetic moment of neighboring Ni atoms. The calculated magnetic moments of Ni atoms are presented in Figure 5 and Figure 6 as well as in Table 5 (in all cases the configurations with the impurity localized at GB are considered).

In case of a clean grain boundary (Figure 5a), we mentioned the oscillations in magnetic moments of Ni atoms in the previous chapter; they are enhanced in the GB region. This enhancement is in agreement with literature [2,33,34,49,50,54], the maximum magnetic moment was found in layer 2.

Further, let us discuss the results concerning the magnetism of GBs with impurities. Here, the “magnetic-moment-decreasing” effect of impurity is suppressing the “magnetic-moment-increasing” effect of grain boundary. In case of GB-Ni_118_Si_2_^(s)^ and GB-Ni_118_Al_2_^(s)^ (Figure 5b) supercells, there is a very similar trend in the distribution of magnetic moments. However, the “magnetic-moment-decreasing” effect is a little bit stronger for substitutional silicon than for substitutional aluminum. This effect is most obvious for atoms in the 2nd (the strongest effect), 3rd and 4th layer. The magnetic moments in the 5th layer are still a little bit below the bulk value. Then, the moments of atoms from further (6th–8th) layers oscillate around the bulk value. In case of the structure GB-Ni_120_Si_2_^(i)^ (Figure 5c), the largest decrease in magnetic moment can be observed near to the impurity. The reason for this is the small free volume caused by interstitial impurity. Here, the magnetic moment distribution is different from the case of structures discussed previously. In particular, the atoms at the boundary are strongly influenced by the interstitial impurity.

The magnetic moments in structures containing GB and impurity exhibit a scattered character in the same layer. A more detailed study reveals that they increase rather monotonically with the distance from the impurity atom at the GB (Figure 6), which gives rise to scattering as Ni atoms in the same layer have different distances from the impurity. For more distant planes, the magnetic moment of Ni atoms converges to the bulk value. The only case where the magnetic moments are increased is that of the planes without impurity atoms in the part of the GB(Ni,Ni). In this case, the Ni atoms do not have any impurity in their neighborhood and therefore the “magnetic-moment-increasing” effect of grain boundary prevails. In Figure 6, the scatter of values corresponding to atoms with the same distance from X is caused by their different distance from GB.

Table 5 presents more details on the nearest Ni atoms of studied impurities. The magnetic moments of Ni atoms lying in the nearest neighborhood of substitutional Al atom (about 0.528-0.588 μ_B_) are lower in comparison to the *μ* of atoms lying in the same layers but further from impurity. The values for Ni atoms more distant from Al are shown in Figure 6 and their magnetic moments are higher than 0.6 μ_B_.

The trends of magnetic moments of Ni atoms in both structures with substitutional impurities are similar. The lowest magnetic moment is found at the nearest Ni atoms. The further two Ni atoms reveal the increase of magnetic moment as they lay in the 2nd layer and are in the vicinity of GB. The last (4th) Ni atoms possess the lowered magnetic moments again as they are accommodated in the 4th layer.

In case of the structure with interstitial Si, the situation is different. The magnetic moment of Ni increases (with small deviation for the third Ni atom) with increasing distance from Si atom (column 3 of Table 5) and it is simultaneously approaching to the GB (column 5 of Table 5).

From the previous studies, it is apparent that the segregation of nonmagnetic impurities has a detrimental effect on GB magnetism [2,56]. Another example of a reduction of magnetic moments due to impurities and other structural imperfections may be found in the paper [57].

Therefore, it may be supposed that the effect of impurities on magnetic properties will be more significant in materials with high GB concentrations, e.g. in nanocrystalline materials. It was demonstrated that nanocrystalline Ni does not exhibit any substantial change in the saturation magnetization compared to a polycrystalline sample [58] but, probably, because of exposition to air, the saturation magnetization is lowered due to a GB contamination by oxygen. The effect of segregation of nonmagnetic elements on the magnetic properties of GBs was found experimentally for tungsten segregating at GBs of nanocrystalline Ni in Reference [59]. Some other works dealing with magnetism on GBs in nanomaterials were also published, e.g. in Reference [60] dealing with Fe. To the best of our knowledge, no such study was made for nanocrystalline Ni with Al or Si.

Comparing the magnetic moment of bulk Ni atom calculated by Všianská and Šob [2] and published in this work, the magnetic moment of Ni atom presented in this work is by 2% larger. For the structure GB-Ni_120_, the differences in magnetic moments are not larger than 2%. There are larger differences in magnetic moments of atoms in structures GB-Ni_118_Al_2_^(s)^, GB-Ni_118_Si_2_^(s)^ and GB-Ni_120_Si_2_^(i)^ calculated in [2] and presented in this work.

The largest differences can be observed for magnetic moments of Ni atoms in the layers GB, 2, 3, 4 and for magnetic moments of impurities. In these layers, the magnetic moments of Ni atoms calculated in this work are always larger than the magnetic moments calculated in Reference [2]. For structures containing substitutional Al and Si the differences are 34% and 54% for the 3rd layer. For the structure GB-Ni_120_Si_2_^(i)^, the difference in magnetic moment of Ni atoms attains its maximum for Ni atoms at the GB layer, the value is almost 93%. 

The above-mentioned differences are caused by the differences in calculations discussed in the section devoted to the interlayer distances and, of course, by a different concentration of impurities at GBs.

According to [61], the solubility of Al in bulk Ni is about 7 at.% at 400 °C, our concentration in bulk and in structure with GB is equal to 1.67 at.%. In [62], the solubility of Si in bulk Ni is found to be 10 at.% at 700 °C. Our concentration is 1.64 at.% in GB-Ni_120_Si_2_^(i)^, 0.83 at.% in Ni_120_Si^(i)^ and 1.64 at.% in Ni_60_Si^(i)^. Despite the temperature effect, our concentrations are lower than the experimental ones and hence, according to Lejček et al. [63], we can consider our values of segregation energies reliable.

### 3.4. Grain Boundary with Impurities and Vacancies

In this chapter, the effect of vacancy in the structures GB-Ni_120,_ GB-Ni_118_Al_2_^(s)^, GB-Ni_118_Si_2_^(s)^ and GB-Ni_120_Si_2_^(i)^ is analyzed. The interactions between the grain boundary, the impurity and the vacancy are studied as well as the effect of the distance between vacancy and impurity atom at the GB. These phenomena are investigated from both the energetic and structural point of view.

#### 3.4.1. Structure Arrangement

In case of impurities, the presence of vacancies is crucial for their diffusion as they enable an easier exchange of atomic positions. In Table 2, the preference of Al and Si to bind to vacancies in bulk Ni material was presented, which further emphasizes the importance of vacancy diffusion mechanism of these two impurities in Ni. In principle, there is a large variety of possible impurity-vacancy configurations at the studied GB (or in its nearest vicinity) which can be investigated.

Here, the vacancies on GBs without impurities are studied at first. In the bulk material, the presence of a vacancy can be characterized by a free volume. This volume can disappear only when the vacancy meets the interstitial atom or another lattice defect, e.g. GB. In this case the vacancies behave unusually [64,65,66], i.e. they can be delocalized or become instable [65]. The volume of delocalized vacancy disappears by rearrangement of neighboring atoms. On the other hand, the instability is related to the movement of the vacancy to another site where the delocalization can occur again.

In this part of study, we have investigated all four above mentioned configurations with vacancies accommodated in various layers. The results concerning the vacancy formation are summarized in Table 6.

**Figure 7:**
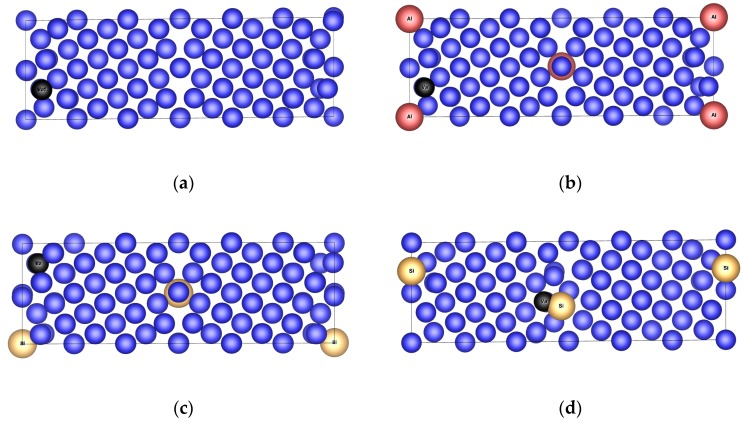
The structures with the lowest energies: (**a**) GB-Ni_119_+Va^L2^, (**b**) GB-Ni_117_Al_2_^(s)^+Va^L2^, (**c**) GB-Ni_117_Si_2_^(s)^+Va^L2^, (**d**) GB-Ni_119_Si_2_^(i)^+Va^L3^. Here, the denoted positions of impurities correspond to the general positions shown in Figure 1b. The dark particles denote the positions of vacancies.

For GB-Ni_119_+Va, GB-Ni_117_Al_2_^(s)^+Va and GB-Ni_117_Si_2_^(s)^+Va (GB-Ni_119_Si_2_^(i)^+Va) structures, the configuration with vacancy in layer 2 (layer 3) reveals the highest decrease of the volume per unit cell and the lowest but still positive energy of vacancy formation. It can be said that the largest decrease in volume per unit cell always corresponds to the most easily but still unfavorably formed vacancy and to the most stable arrangement with the lowest energy of formation with respect to the standard element reference states (Table 7). However, as all structures possess the positive values of the energy of formation they must be considered as unstable at 0 K. The structures with the lowest energies are shown in Figure 7.

In case of the structure GB-Ni_119_+Va^L2^, there occur changes in positions of atoms in the closest neighborhood of the vacancy, as they tend to occupy the free space provided by this lattice defect. The vacancy even attracts the Ni atom on the opposite side of the grain boundary, which corresponds to the vacancy mirror image.

The vacancy in the GB-Ni_117_Al_2_^(s)^+Va^L2^ and GB-Ni_117_Si_2_^(s)^+Va^L2^ structure also causes the attraction of Ni atoms lying in the nearest GB layer towards the vacancy site. The effect is largest for the Ni atoms with the same *z* coordinate as the vacancy.

The vacancy in the structure GB-Ni_117_Al_2_^(s)^+Va^L2^ attracts the Ni atom situated on the opposite side of the GB and in the same plane as vacancy (*z* = 0.0). In the lowest-energy structure, this atom is situated almost at the GB. This effect is not as strong as in the case of GB-Ni_117_Si_2_^(s)^+Va^L2^ structure but it is still very significant. As a consequence of this structural change, another two Ni atoms are repulsed by the approaching Ni atom. Their *z* coordinates are changing from the value of 0.75 to 0.77 and from 0.25 to 0.23 to make the space for the newcomer. Here, the Al atom closer to the vacancy is also slightly moving towards the vacancy.

In the structure GB-Ni_117_Si_2_^(s)^+Va^L2^ with the vacancy in the second layer, the largest structure change occurs in the plane with *z* = 0.75. This is the plane where the vacancy is placed, i.e., in the plane next to the plane with impurity. Here, the most affected Ni atom is situated in the second layer on the opposite side of the grain boundary with respect to the vacancy. This atom moves towards the grain boundary. In the lowest-energy structure, this atom is located almost at the grain boundary. This position rearrangement causes the repulsion of the Ni atom on the grain boundary (*z* = 0.75). The final position of coming Ni atom causes other changes in the structure arrangement of Ni atoms in the plane *z* = 0.5. The Si atom closer to the vacancy is also affected. This atom is changing its *z* coordinate from *z =* 0 to *z =* 0.19.

The vacancy in the structure GB-Ni_119_Si_2_^(i)^+Va^L3^ causes the largest changes in the arrangement of atoms. The free volume of vacancy is partially occupied by the interstitial Si atom that is situated in the same plane as the vacancy (*z* = 0.75). This means that the interstitial Si in the vicinity of the vacancy changes its position and moves away from the grain boundary towards the bulk region. Its equilibrium position is between the GB and the 2nd layer. The second most affected atom is the Ni atom located in the second layer and in the same plane as vacancy and silicon impurity. This atom is also moving towards the position where the vacancy is.

Despite of the changes in the arrangement of the atoms in the lowest-energy structures, it can be concluded that the existence of the vacancy is preserved and no delocalization or instability has been observed.

**Figure 8:**
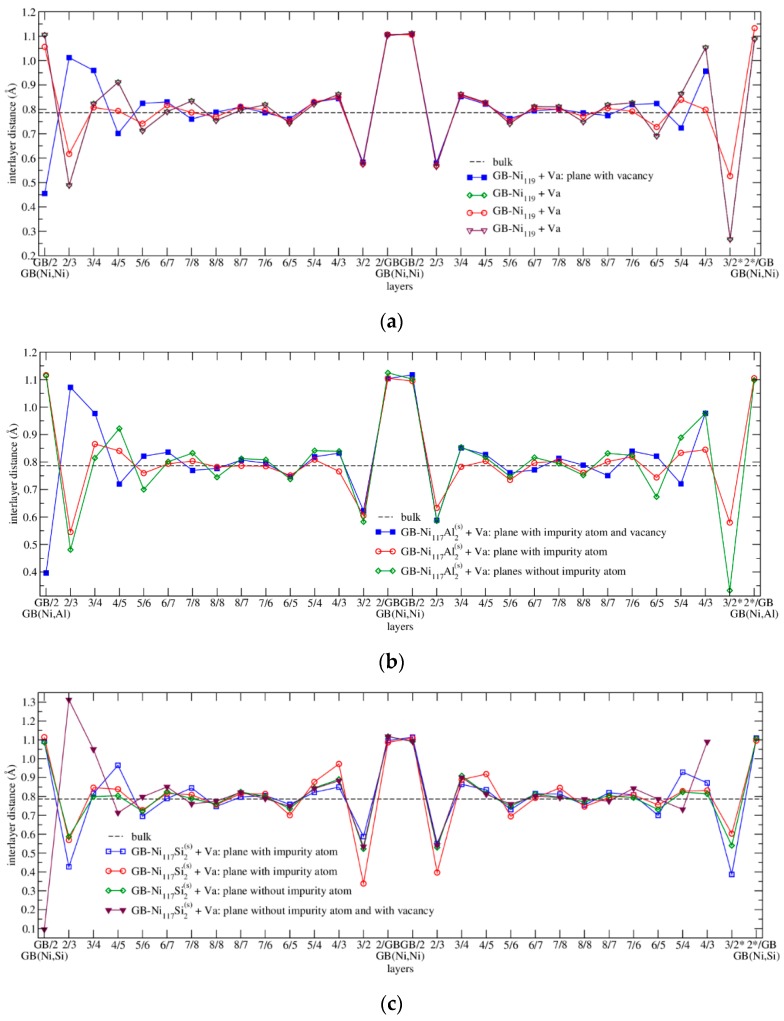

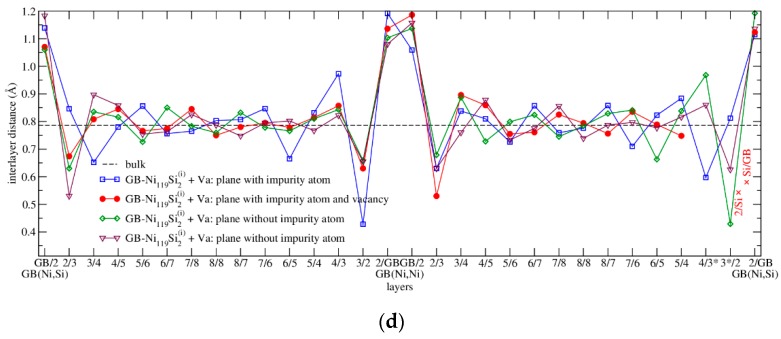
Interlayer distances in supercells with the GB, impurity and vacancy: (**a**) GB-Ni_119_+Va^L2^, (**b**) GB-Ni_117_Al_2_^(s)^+Va^L2^, (**c**) GB-Ni_117_Si_2_^(s)^+Va^L2^, (**d**) GB-Ni_119_Si_2_^(i)^+Va^L3^. The superscripts ^L2^ and ^L3^ denote the position of the vacancy in the second and third layer, respectively. × indicates the distances of interstitial Si in the layer with vacancy, which changed its position due to the structure relaxation. * denotes the layer with the vacancy. The dashed line corresponds to the interlayer distance in ferromagnetic *fcc* Ni (this work). The lines do not reflect any physical meaning; they are used to guide the eye only. For a better demonstration of results in the cases of Figures (b–d), the plane with impurity atom is always presented so that the impurity is on the edges of the figures. This was achieved by the shift of ½ of the lattice parameter *b* of lines representing the results of some planes with impurities (the middle plane of GB-Ni_118_Al_2_^(s)^ and GB-Ni_118_Si_2_^(s)^ or the second plane of GB-Ni_120_Si_2_^(i)^) and one without impurity (the middle plane of GB-Ni_120_Si_2_^(i)^).

The interlayer distances in all mentioned structures were also analyzed and the results are presented in Figure 8.

The situation in GB-Ni_119_+Va^L2^ structure is the least complicated as this structure does not contain any impurity. The planes which do not contain the vacancy (green line with diamonds, red with circles and violet with triangles in Figure 8a, where the green and violet line are overlapping) reveal the typical shape of the curve describing the dependence of the interlayer distances on the type of layer. There is a large gap between the GB and second Ni layer (GB/2) followed by reduced spacing between layers 2 and 3 (2/3 and 3*/2). Some oscillations may be found in the interlayer distances between further layers, which converge to the interlayer distance typical for the bulk material. The reason why these curves are not ideally symmetric with respect to the center of the figure is that the interlayer distances are influenced by vacancy even if it is not placed directly in these planes. The plane in GB-Ni_119_+Va^L2^ structure, which contains the vacancy (blue line with squares in Figure 8a), reveals untypical behavior. The Ni atom placed in the 2nd layer on the opposite side of the GB than vacancy (the mirror image of the vacancy) moves towards the GB in comparison with the plane without the vacancy and hence the value of interlayer distance GB/2 is very low. This shift is compensated by the increase of the interlayer distances 2/3 and 3/4 in the left part of Figure 8a. The missing points on the right-hand side of the curve correspond to the vacancy. The presence of the vacancy also causes the scatter of interlayer distances on the other curves at the edges of the figure and, as there are no impurities, the curves overlap in the middle.

The planes in GB-Ni_117_Al_2_^(s)^+Va^L2^ structure which do not contain the vacancy (green with diamonds, describing behavior in two planes *z =* 0.25 and 0.75 and red line with circles) in Figure 8b reveal the same trends as described for the structure GB-Ni_119_+Va^L2^. The plane in the GB-Ni_117_Al_2_^(s)^+Va^L2^ structure which contains the vacancy and impurity (blue line with squares in Figure 8b) reveals untypical behavior. The Ni atom placed in the 2nd layer on the opposite side of the GB than the vacancy (the mirror image of the vacancy) moves towards the GB by 0.72 Å in comparison with the plane without vacancy. This shift is compensated by the increase of the interlayer distances 2/3 and 3/4 in the most left part of the Figure 8b. The missing points on the right-hand side of the curve correspond to the vacancy.

The case of the GB-Ni_117_Si_2_^(s)^+Va^L2^ structure is similar to GB-Ni_117_Al_2_^(s)^+Va^L2^, i.e. the planes without a vacancy (blue line with squares, green with diamonds and red with circles in Figure 8c) reveal the typical shape of the curves. The plane which contains the vacancy (violet line with triangles in Figure 8c) reveals the increase of the interlayer distances 2/3 and 3/4 in the most left part of the Figure 8c which is caused by the attraction of the Ni atom in the 2nd layer on the opposite side of GB (the mirror image of the vacancy) towards the vacancy. From this reason, the most left violet point possesses the lowest value of interlayer distance. The influence of the vacancy on the structure is much higher in this structure than in the structure containing aluminum which is demonstrated by a higher scatter of the points on the left-hand part of curves describing the planes with the vacancy.

In case of the GB-Ni_117_Si_2_^(i)^+Va^L3^ structure, the situation is even more complicated, in particular in the case of the plane with an impurity and a vacancy (red line with circles in Figure 8d). On the right-hand side of this line, there are two points missing which correspond to the vacancy and there are two points × added which denote the distances of the Si atom from the GB and the 2nd layer. From their position, it is obvious that the Si atom moved away from the GB towards the 2nd layer. The green curve with diamonds and violet one with triangles in Figure 8d reveal typical trends with a maximum of the distance between the GB and layer 2 (GB/2) and a minimum of the distance between the layer 2 and 3 (2/3).

#### 3.4.2. Energetics

The energy of formation of the structures with impurity, vacancy and GB with respect to the standard element reference states is evaluated using Equation (8):

(8) $$E^{f}\left(GB-Ni_{m}Al_{n}Si_{o}+Va\right)=E_{GB-Ni_{m}Al_{n}Si_{o}+Va}-\left(mE_{1fccNi}+nE_{1fccAl}+oE_{1diamSi}\right).$$

Here, $E_{GB-Ni_{m}Al_{n}Si_{o}+Va}$ is the total energy of the structure with *m* Ni atoms, *n* Al atoms or *o* Si atoms, GB and one vacancy. The terms $E_{1fccNi}$, $E_{1fccAl}$ and $E_{1diamSi}$ correspond to the total energy of one atom in its standard element reference state. The obtained results are summarized in Table 7.

According to the data in Table 7, we can see that the energy of formation of the structure with a vacancy and a GB with respect to the standard element reference states is the lowest for structures where the vacancy is situated in the second layer for GB-Ni_119_+Va, GB-Ni_117_Al_2_^(s)^+Va, and GB-Ni_117_Si_2_^(s)^+Va and in the third layer for GB-Ni_119_Si_2_^(i)^+Va. The position of the vacancy has the effect on the energy of formation up to 20%. Because of the positive values of $E^{f}\left(GB-Ni_{m}Al_{n}Si_{o}+Va\right)$, all structures must be considered as unstable at 0 K. Nevertheless, we can expect their existence at higher temperatures when also the simultaneous existence of more configurations of one structure having vacancies in various positions is probable as the energy differences between them are very small. In such a case, we expect the Si^(i)^ to be more stable than Si^(s)^. The lowest-energy configurations for structure GB-Ni_117_Al_2_^(s)^+Va, GB-Ni_117_Si_2_^(s)^+Va and GB-Ni_119_Si_2_^(i)^+Va were used for the calculation of following quantities.

The key quantity for the vacancy formation energy is the difference of total energy for the defective (*E_GB+Va_*) and perfect (*E_GB_*) systems both in relaxed state [67]. Structural relaxation can reduce the vacancy formation energy by more than 30%. It is therefore important to include structural relaxation when calculating vacancy formation energies from first principles. Vacancies, created at the surface or at defects, exist at a certain concentration in all materials, depending exponentially on the formation energy of the vacancy and the temperature [68].

The stability of vacancy may be characterized by the vacancy formation energy which can be calculated according to the following Equation (9):

(9) $$E^{f}\left(Va\right)=\left(E_{GB+X+Va}+E_{1fccNi}\right)-E_{GB+X}.$$

Here, $E_{GB+X+Va}$ and $E_{GB+X}$ is the total energy of *fcc* Ni with GB, impurity X and Va and of *fcc* Ni with GB and impurity X. The value of *E^f^(Va)* provides the information whether there is a tendency towards the vacancy formation. The data obtained using Equation (9) are listed in Table 6.

In all configurations of studied structures, the energy of vacancy formation is positive, which means that the vacancies destabilize the structure. In a case of GB-Ni_119_+Va structure, the lowest-energy configuration is that one with the vacancy situated in the 2nd layer (see Table 7). In this structure, the vacancy reveals the lowest positive energy of formation 0.5791 eV.Va^−1^, i.e. is less unstable than in the other structures with the same composition. For this structure, we can also observe the largest decrease in the volume −9.7127 Å^3^.Va^−1^. The vacancy in the 2nd layer exhibits also the lowest energy for the GB-Ni_117_Al_2_^(s)^+Va and GB-Ni_117_Si_2_^(s)^+Va structures where the energy of vacancy formation is 0.6169 eV.Va^−1^ and 0.2466 eV.Va^−1^, respectively. The formation volume is −3.3396 Å^3^.Va^−1^ and −5.7481 Å^3^.Va^−1^. In the case of the last structure GB-Ni_119_Si_2_^(i)^+Va, the lowest-energy configuration is that one with a vacancy situated in the 3rd layer. Here, the vacancy formation energy is 0.5746 eV.Va^−1^ and volume decrease is −14.3407 Å^3^.Va^−1^.

From the comparison of energies of vacancy formation in GB-Ni_119_+Va, GB-Ni_117_Al_2_^(s)^+Va, GB-Ni_117_Si_2_^(s)^+Va and GB-Ni_119_Si_2_^(i)^+Va configurations, it is obvious that the vacancy formation energy is the lowest for the structure GB-Ni_117_Si_2_^(s)^+Va^L2^ (0.2466 eV.Va^−1^). However, we have to take into account that the vacancy formation is energetically unfavorable process in all mentioned cases because the *E^f^(Va)* values are always positive. It can be said that the largest decrease in volume per atom always corresponds to the lowest-energy configuration. From all four configurations, the largest decrease was observed for the structure GB-Ni_119_Si_2_^(i)^+Va^L3^ (14.3407 Å^3^.Va^−1^).

We have not found any literature data about the energetics of the Va formation at GBs in *fcc* Ni. The energies of vacancy formation at the Σ3(111) GB in vanadium were calculated in Reference [69]. Here, the formation energy of a vacancy at the GB is 2.51 eV, in the second layer 0.18 eV, in the third layer 1.65 eV and 2.54 eV in the fourth layer. It is in a good agreement with experimental data of 1.8–2.6 eV [70]. This trend in formation energy of vacancy with respect to its position is similar to the trend in vacancy formation energy for Σ5(210) GB in *fcc* Ni found in this work.

We can also evaluate further energy-related quantities, which are described in the following text and defined using Equations (10)–(15). The first such quantity is the binding energy of a vacancy to couple of lattice defects GB+X (the tendency of vacancy to move from the bulk to the GB with impurity) which is evaluated as:

(10) $$E^{b}\left(Va;GB+X\right)=\left(E_{GB+X+Va}+E_{bulk}\right)-\left(E_{bulk+Va}+E_{GB+X}\right).$$

Here, $E_{bulk+Va}$ is the total energy of bulk *fcc* Ni with a vacancy. Based on the value of $E^{b}\left(Va;GB+X\right)$, we can predict whether the couple of defects GB+X facilitates the formation of vacancies in comparison with the bulk material. When we simplify the Equation (10) to the form:

(11) $$E^{b}\left(Va;GB\right)=\left(E_{GB+Va}+E_{bulk}\right)-\left(E_{bulk+Va}+E_{GB}\right),$$

we get the binding energy of the vacancy to the clean GB, which is -0.831 eV for the most stable configuration GB-Ni_119_+Va^L2^.

We can also evaluate the binding energy of impurity to couple of lattice defects GB+Va (the tendency of impurity to move from the bulk to the GB with vacancy) as:

(12) $$E^{b}\left(X;GB+Va\right)=\left(E_{GB+X+Va}+E_{bulk}\right)-\left(E_{bulk+X}+E_{GB+Va}+\frac{E_{GB+X}}{2}-\frac{E_{GB}}{2}\right).$$

Further, we can also study how two defects interact in the presence of the third one. In our research, we can find three such situations. The first one is the vacancy-impurity interaction at the Σ5(210) GB described with the help of the vacancy-impurity binding energy at GB. This quantity tells us how the GB simplifies or complicates the formation of X+Va couple from the separated X and Va both being at the GB. Here, the vacancies as well as impurities at the GB are used as a reference state. The vacancy-impurity binding energy is described for both the substitutional or interstitial impurity as follows:

(13) $$E^{b}\left(GB+X;GB+Va\right)=\left(E_{\mathrm{GB}+X+Va}+E_{GB}\right)-\left(E_{GB+X}+E_{GB+Va}\right).$$

Here, $E_{GB+Va}$ is the total energy of bulk *fcc* Ni with GB and vacancy and the term $E_{GB}$ is used to keep the balance in number of GBs and Ni atoms employed.

The vacancy-GB binding energy at the presence of impurity can be calculated using Equation (14):

(14) $$E^{b}\left(X+Va;X+GB\right)=\left(E_{GB+X+Va}+E_{bulk+X}\right)-\left(E_{bulk+X+Va}+E_{GB+X}\right).$$

This energy provides the information how does the impurity simplify or complicate the formation of GB+Va couple from the separated GB and Va, in other words how simple the transfer of Va to GB in the presence of X in both places is.

The final (third) possibility of the above-mentioned interactions of two defects in the presence of the third one is characterised by the X-GB binding energy at presence of Va, which is defined by Equation (15):

(15) $$E^{b}\left(Va+X;Va+GB\right)=\left(E_{GB+X+Va}+E_{bulk+Va}\right)-\left(E_{bulk+X+Va}+E_{GB+Va}\right).$$

This equation describes how does the Va simplify or complicate the formation of GB+X couple from the separated GB and X (i.e. the transfer of X to GB) in the presence of Va in both places. The data obtained by means of Equations (10) and (12)–(15) are summarized in Table 8.

According to this table, it can be said that the binding energy of vacancy to couple of GB+X (*E^b^(Va;GB+X)*) is negative for all structures GB-Ni_117_Al_2_^(s)^+Va^L2^, GB-Ni_117_Si_2_^(s)^+Va^L2^ and GB-Ni_119_Si_2_^(i)^+Va^L2^. This means that the vacancy tends to stay together with the impurity and GB. The highest released binding energy corresponds to the structure GB-Ni_117_Si_2_^(s)^+Va^L2^ (−1.18 eV) and the lowest released energy is found for the structure GB-Ni_117_Al_2_^(s)^+Va^L2^ (−0.85 eV).

Considering the second column of Table 8, the binding energy of impurity to couple of lattice defects GB+Va (*E^b^(X;GB+Va)*), we can observe negative values for all structures GB-Ni_117_Al_2_^(s)^+Va^L2^, GB-Ni_117_Si_2_^(s)^+Va^L2^ and GB-Ni_119_Si_2_^(i)^+Va^L2^. The maximum released energy is found for structure GB-Ni_119_Si_2_^(i)^+Va^L3^ (−8.98 eV). It means that the tendency of interstitial Si to move from the bulk to the GB with a vacancy is very strong. The minimum released value (−0.10 eV) corresponds to the structure GB-Ni_117_Al_2_^(s)^+Va^L2^.

When considering the values of vacancy-impurity binding energy at the GB (*E^b^(GB+X;GB+Va)*) summarized in the 4th column of Table 8, it can be concluded that the GB-Ni_117_Si_2_^(s)^+Va^L2^ exhibits the highest released energy (−0.33 eV) which means that these two defects tend to stay together. The structure GB-Ni_119_Si_2_^(i)^+Va^L3^ exhibits positive value of this quantity (0.04 eV). Hence, the impurity and the vacancy binding are not preferred in this structure. In case of the GB-Ni_119_Si_2_^(i)^+Va^L3^_,_ there is neither tendency for binding nor splitting of vacancy and impurity at the GB as the binding energy is zero. Comparing the binding energy *E^b^(X;Va)* of Ni_58_Al^(s)^+Va and Ni_58_Si^(s)^+Va mentioned in Table 2 (being for the most stable structures -0.0451 and −0.1110 eV.X^−1^Va^−1^, respectively) with *E^b^(GB+X^(^*^s/i*)*^*;GB+Va)* in GB-Ni_117_Al_2_^(s)^+Va^L2^ and GB-Ni_117_Si_2_^(s)^+Va^L2^ provided in Table 8 (being 0.04 and −0.33 eV.X^−1^Va^−1^), we can say that the vacancy and impurity are coupled stronger in case of substitutional Si than in structure with substitutional Al. However, no particular tendency was observed for the binding between a vacancy and an impurity when comparing the structures with and without GB. In case of the Al impurity, the GB causes the weakening of the binding energy (positive value 0.04 eV.X^−1^Va^−1^) which results even in the preference of splitting of these two defects. In case of the substitutional Si, the tendency is reverse, i.e. the GB causes the enhancing of the binding energy.

The values of the vacancy-GB binding energy in the presence of X (*E^b^(X+Va;X+GB)*) in the fifth column in Table 8 show how the impurity simplifies or complicates the formation of GB+Va couple from the separated GB and Va. In all studied cases, the coupling of Va and GB is favorable as this binding energy is negative. The highest released energy (−1.05 eV.Va^−1^GB^−1^) corresponds to the structure GB-Ni_117_Si_2_^(s)^+Va^L2^. On the other hand, the lowest binding energy (−0.75 eV.Va^−1^GB^−1^) was found for the structure GB-Ni_117_Al_2_^(s)^+Va^L2^.

When considering the X-GB binding energy in the presence of Va (*E^b^(Va+X;Va+GB)*), (the sixth column in Table 8), we can observe that X can be transferred to GB most easily in the structure GB-Ni_119_Si_2_^(i)^+Va^L3^ because of the negative binding energy (−3.52 eV.X^−1^GB^−1^). On the other hand, the maximum value was obtained for structure GB-Ni_117_Si_2_^(s)^+Va^L2^ (0.02 eV.X^−1^GB^−1^).

It is not necessary to analyze the separate defects only. Their couples can be studied too. The energy of formation of couple defect (X+Va) at the GB from the cell with GB, X in SER state and pure vacancy is, for a substitutional impurity, calculated as follows:

(16) $$E^{f}\left(X+Va\right)=\left(E_{GB-Ni_{m}X^{\left(s\right)}{}_{n}+Va}+\left(n+1\right)E_{1fccNi}\right)-\left(E_{GB-Ni_{m+n+1}}+E_{1SERX}+E_{1Va}\right),$$

and for an interstitial impurity as:

(17) $$E^{f}\left(X+Va\right)=\left(E_{GB-Ni_{m}X^{\left(i\right)}{}_{n}+Va}+E_{1fccNi}\right)-\left(E_{GB-Ni_{m+1}}+E_{1SERX}+E_{1Va}\right).$$

In the last two Equations (16) and (17), the energy of a vacancy without atoms is considered to be $E_{1Va}=0\;\mathrm{eV}.\mathrm{Va}$^−1^. The reason for including this quantity in these equations is purely educational to keep them consistent and complete with respect to the theory. The values of $E^{f}\left(X+Va\right)$ can be interpreted as energies needed/released when the impurity X and Va enter the GB in *fcc* Ni to form a X+Va couple and the calculated values are listed in the second column of Table 9.

In this column, we can observe that all formation energies *E^f^(X+Va)* at the GB acquire quite high negative values. It means that the integration of X+Va couple into the structure with GB is energetically favorable in all three structures. The couple X+Va can incorporate most easily into the structure GB-Ni_119_Si_2_^(i)^+Va^L3^. It is because of the negative formation energy of X+Va which equals to −1.27 eV.X^−1^Va^−1^. This tendency is lower by 15% for the structure GB-Ni_117_Al_2_^(s)^+Va^L2^ and by 12% for GB-Ni_117_Si_2_^(s)^+Va^L2^.

The binding energy of triple defect (GB;X;Va) with respect to the total energy of three single defects (GB, X and Va) (*E^b^(X;GB;Va)*) was calculated according to the following equation:

(18) $$E^{b}\left(GB;X;Va\right)=\left(E_{GB+X+Va}+E_{bulk}\right)-\left(E_{GB}+E_{bulk+X}+E_{bulk+Va}\right).$$

The results are summarized in Table 9. Because of the negative values of $E^{b}\left(GB;X;Va\right)$, it was concluded, that these three defects (GB, impurity, vacancy) prefer to bind in one structure. The maximum value of $E^{b}\left(GB;X;Va\right)$ (−4.35 eV.X^−1^Va^−1^GB^−1^) corresponds to the structure GB-Ni_119_Si_2_^(i)^+Va^L3^, the binding energies of GB-Ni_117_Al_2_^(s)^+Va^L2^ and GB-Ni_117_Si_2_^(s)^+Va^L2^ are rather lower (both −0.92 eV.X^−1^Va^−1^GB^−1^).

Similarly, the binding energy of triple defect (GB;X;Va) with respect to the total energy of couple defects (GB+X, GB+Va and X+Va) (E^b^(X;GB;Va)*) was calculated according to Equation (19):

(19) $$E^{b}{\left(GB;X;Va\right)}^{*}=\left(E_{GB+X+Va}+E_{bulk}\right)-\left(E_{GB+X}+E_{GB+\mathrm{Va}}+E_{bulk+X+Va}\right).$$

The results are listed in Table 9. According to the values obtained from Equation (19), the triple defects can be considered as unstable with respect to the couple defects in the structure GB-Ni_119_Si_2_^(i)^+Va^L3^ structure because of their positive binding energy (0.31 eV.X^−1^Va^−1^GB^−1^). On the other hand, the stable triple defect can be found in the structure GB-Ni_117_Al_2_^(s)^+Va^L2^ where the binding energy reveals negative value.

In principle, the theoretical total energy of studied structures ($E_{GB+X^{\left(s/i\right)}+Va}^{theo}$) can be calculated from separated contributions: total energy of bulk Ni and X, total energies of formation of particular defects and total energies of binary and ternary interactions of these defects. For the most complicated structure containing all three defects GB, X and Va, this procedure is described by Equation (20):

(20) $$E_{GB+X^{\left(s/i\right)}+Va}^{theo}=E_{nfccNi}+1E_{1SERX}+E_{GB}^{f}+E_{X}^{f}+E_{Va}^{f}+E_{GB;X}^{b}+E_{GB;Va}^{b}+E_{X;Va}^{b}+E_{GB;X;Va}^{b}.$$

The data calculated according to this equation are summarized in Table 10.

Furthermore, the theoretical energies $E_{GB+X^{\left(s/i\right)}+Va}^{theo}$ can be compared with the total energies of particular structures as follows:

(21) $$E^{diff}=E_{GB+X^{\left(s/i\right)}+Va}^{calc}-E_{GB+X^{\left(s/i\right)}+Va}^{theo}.$$

The results of this comparison are again exhibited in Table 10. In case that all contributions in $E_{GB+X^{\left(s/i\right)}+Va}^{theo}$ are calculated properly and no further contribution should be considered, $E^{diff}$ should be zero. In a case of structures with single defect (GB or X or Va) and in a case of structure with two defects (X and Va) the value of $E^{theo}$ and $E^{calc}$ do not differ. In case of structures with all possible combination of defects, the values of $E^{diff}$ are smaller than zero. The lowest value of $E^{diff}$ corresponds to the structure with a GB and substitutional impurity. It means that the fully ab initio calculated values of $E_{GB+X^{\left(s/i\right)}+Va}^{calc}$ are more negative than the theoretical ones, $E_{GB+X^{\left(s/i\right)}+Va}^{theo}$. This can be caused by the fact that the contributions of particular defects and their combinations were calculated from structures at equilibrium volumes which differ from equilibrium volumes of the final structures. The other explanation for this difference could be that there are possible stabilizing interactions in the directly ab initio calculated values in comparison to the theoretical energy obtained from separated contributions.

To make our research on segregation complete, the further three possible types of segregation should be analyzed taking into the account the presence of Va at the GB or in the bulk or in both of these places. Hence, the segregation energy of impurity from the bulk to the GB with Va was evaluated using the following Equation (22):

(22) $$E_{Va^{GB}}^{seg}\left(X\right)=\left(E_{GB+X+Va}-E_{GB+Va}\right)-\left(E_{bulk+X}-E_{bulk}\right)=E_{Xat\left(GB+Va\right)}-E_{Xinbulk},$$

the segregation energy of impurity from the bulk with Va to the GB was calculated according to the Equation (23):

(23) $$E_{Va^{bulk}}^{seg}\left(X\right)=\frac{\left(E_{GB+X}-E_{GB}\right)}{2}-\left(E_{bulk+X+Va}-E_{bulk+Va}\right)=E_{XatGB}-E_{Xin\left(bulk+Va\right)},$$

and, finally, the segregation energy of impurity in the structure with the vacancy both in the bulk and at the GB (impurity-GB binding energy at presence of vacancy) was expressed by Equation (24):

(24) $$\begin{array}{c} E_{Va^{bulk};Va^{GB}}^{seg}\left(X\right)=\left(E_{GB+X+Va}-E_{GB+Va}\right)-\left(E_{bulk+X+Va}-E_{bulk+Va}\right)\\ =E_{Xat\left(GB+Va\right)}-E_{Xin\left(bulk+Va\right)}. \end{array}$$

In these equations, $E_{GB+X+Va}$ and $E_{GB+Va}$ are the energies of the GB+Va structure with and without impurity atom (both containing the same number of Ni atoms) and $E_{bulk+X+Va}$ and $E_{bulk+Va}$ are the total energies of the bulk material containing Va with and without impurity (again, both containing the same number of Ni atoms). The energy difference for bulk Ni material has to be subtracted here to eliminate the energetics of adding an impurity to the bulk material. $E_{XatGB+Va}$ and $E_{Xinbulk+Va}$ are the total energies of the impurity at the GB and in the bulk material, both also containing Va. In the last term of these three equations, the position of impurity in bulk material was always considered as substitutional, because this type of position of impurity is more stable than the interstitial one. After a deeper examination of this issue in Equation (24), it was found that the $E_{Va^{bulk};Va^{GB}}^{seg}\left(X\right)$ quantity is, in case of the substitutional impurity, consistent with the X-GB binding energy at presence of Va, which is described by Equation (15). However, in case of the interstitial impurity, the results differ. The reason for this difference is that we used the bulk material with interstitial Si as the reference state in case of Equation (15) and in case of the Equation (24), the bulk material with the more stable substitutional Si was used.

The results obtained using Equations (7) and (22)–(24) are summarized in Table 11.

According to the values of segregation energies listed in Table 11, we can conclude that the segregation energies are always the most negative for the structures with interstitial impurity. The segregation energies $E_{Va^{bulk}}^{seg}\left(X\right)$ and $E_{Va^{bulk};Va^{GB}}^{seg}\left(X\right)$ (the fourth and the fifth column of Table 11) are almost the same (close to −0.13 eV.X^−1^). These values are much lower than the values listed in the second and third column. This is caused by the presence of the Va in the bulk material which suppress the segregation. The aluminum at the substitutional position also prefers to segregate at the GB because of its negative segregation energy. However, this tendency is lower in comparison with Si^(i)^. The silicon at substitutional position usually prefers to stay in the bulk because of its positive segregation energy except for the case where Va is placed at the GB. In this case Va provides the additional space for the impurity and the segregation energy becomes negative (−0.108 eV.X^−1^).

It can be concluded that the vacancy makes the segregation less desirable in the case of Al^(s)^ and Si^(i)^ except of the case of $E_{Va^{GB}}^{seg}\left(Si^{\left(s\right)}\right)$, where the vacancy supports the segregation. In the remaining cases, the Si^(s)^ prefers to stay in the bulk. 

#### 3.4.3. Magnetism

**Figure 9:**
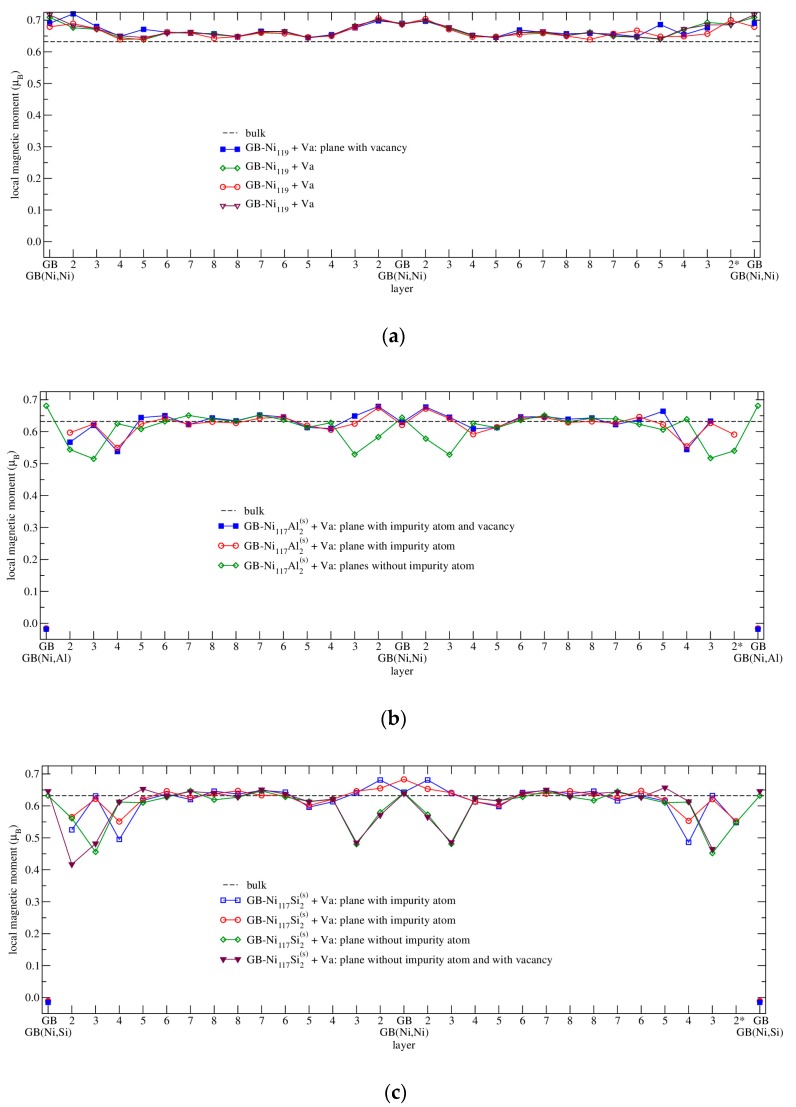

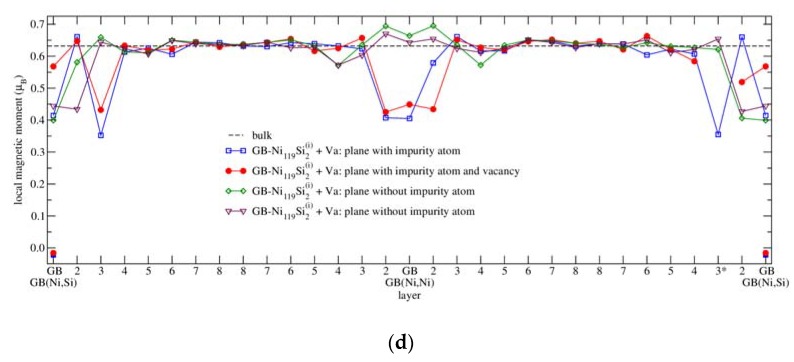
Magnetic moments of Ni atoms in supercells with a GB, an impurity and a vacancy: (**a**) GB-Ni_119_+Va^L2^, (**b**) GB-Ni_117_Al_2_^(s)^+Va^L2^, (**c**) GB-Ni_117_Si_2_^(s)^+Va^L2^, (**d**) GB-Ni_119_Si_2_^(i)^+Va^L3^. The superscripts ^L2^ and ^L3^ denote the position of the vacancy in the second and third layer, respectively. * denotes the layer with the vacancy. The full points mark the magnetic moments of impurities. The dashed line shows the magnetic moment of *fcc* bulk Ni of 0.632 μ_B_ calculated in this work. For a better demonstration of results in Figures (b–d), the plane with impurity atom is always presented so that the impurity is on the edges of the figures. This was achieved by the shift of ½ of the lattice parameter *b* of lines representing the results of some planes with impurities (the middle plane of GB-Ni_118_Al_2_^(s)^ and GB-Ni_118_Si_2_^(s)^ or the second plane of GB-Ni_120_Si_2_^(i)^) and one without impurity (the middle plane of GB-Ni_120_Si_2_^(i)^).

From a comparison of the distribution of magnetic moments in the structure containing a GB and an impurity with the structure containing a GB, an impurity and a vacancy, it is obvious that the vacancy causes the diversification of magnetic moments of Ni atoms. This means that the difference between the magnetic moment per atom of the structure with and without vacancy (Δ*μ_at_*) is non-zero and it acquires both positive and negative values. The scatter in values of magnetic moments of the Ni atom is up to ±0.06 μ_B_. When the vacancy moves from its position at the GB to another layer the region of scatter of magnetic moments moves in the same direction. This effect was observed in structures with all kinds of impurities. Some examples of the distribution of magnetic moments in the structures GB-Ni_119_+Va^L2^, GB-Ni_117_Al_2_^(s)^+Va^L2^, GB-Ni_117_Si_2_^(s)^+Va^L2^ and GB-Ni_119_Si_2_^(i)^+Va^L3^ are shown in Figure 9.

From Figure 9 it is obvious that the distance between the two adjacent GBs is large enough to achieve a bulk-like behavior of magnetic moments between them, i.e. the magnetic moment of *fcc* bulk Ni of 0.632 μ_B_ calculated in this work. Comparing Figure 9a with Figure 9b–d, it can be concluded that the structure without impurities GB-Ni_119_+Va^L2^ (Figure 9a) exhibits a much lower scatter in magnetic moments which occurs only in the nearest vicinity of the vacancy, i.e. at the edges of the figure. On the other hand, the structures with impurities (Figure 9b–d) show a much larger scatter of magnetic moments in the vicinity of impurity. The above-mentioned comparison also shows that the GB and Va cause the increase of magnetic moments, which is demonstrated by the position of all curves in Figure 9a above the value of magnetic moment of *fcc* bulk Ni of 0.632 μ_B_ calculated in this work. On the contrary, the other structures (Figure 9b–d) contain also atoms with magnetic moments lower than that calculated for *fcc* Ni. In case of the GB-Ni_119_+Va^L2^ structure (Figure 9a), atoms located at clean GB in different planes but in the same layer have the same magnetic moment (overlapping curves in the middle of figure) which is much higher than that calculated for *fcc* Ni. The maximum of magnetic moment of 0.72 μ_B_ was reached for atoms in the layer 2. Similar behavior was not observed for structures with impurities which cause the decrease and a large scatter of magnetic moments even at the clean GB.

In case of the structure GB-Ni_117_Al_2_^(s)^+Va^L2^, the GB with a vacancy enables the Ni atoms to migrate towards the free space of the vacancy and hence to decrease their distance from Al atoms. This change of position causes only a minor decrease of magnetic moments (hundredths of μ_B_) and their mild scattering. The differences in magnetic moments between the two planes with impurity are not very large, as it can be seen from Figure 9b—blue line with squares and red with circles. In case of the planes with *z =* 0.25 and 0.75 (Figure 9b—green line with diamonds), the magnetic moments are even identical. The symmetry of curves with respect to the center of the figure proves that the magnetic moments do not depend dominantly on the position of the vacancy (layer denoted by *) which would cause the asymmetry close to this layer. By other analyses, it was found that the dominating effect is the distance from the impurity, which is different for different atoms in the same layer. This causes the differences of the shape of curves in both the marginal and central part of the figures. In the central part of the figures labelled as GB(Ni,Ni), this effect is visible because of the shift of some lines representing the results of planes with impurities in the middle of the supercell (see the label of Figure 4 and Figure 9). Then the atoms situated in some plane at the GB without impurity GB(Ni,Ni) can be very close to the impurity that is placed in some plane of GB(Ni,X) and can be influenced by them. In case of the planes without the impurity, the value of the magnetic moment at the GB exactly correlates with the distance from the impurity, i.e. the higher distance the higher magnetic moment.

The behavior of magnetic moments in GB-Ni_117_Si_2_^(s)^+Va^L2^ is similar to that in GB-Ni_117_Al_2_^(s)^+Va^L2^. The Ni atoms in the vicinity of the vacancy change their magnetic moments in dependence on the distance from the impurity. The differences between the two planes with impurities (Figure 9c—blue curve with squares and red with circles) are not very large. They reveal the same trends. The same behavior is observed for planes without impurity (Figure 9c—green curve with diamonds and violet with triangles). The couples of planes even reveal similar trends in the vicinity of the vacancy (the right part of the figure denoted by *). The only case when they differ is the opposite part of the GB with respect to the position of the vacancy (the left-hand side of the figure), where the Ni atoms in the plane of the vacancy migrate towards the vacancy and at the same time towards the impurity, which causes the decrease of the magnetic moment on the violet curve with triangles in the 2nd layer and bigger scatter in magnetic moments of other atoms as their positions are also slightly influenced.

Comparing the effects of substitutional impurities (Figure 9b,c), it can be concluded that Si causes a larger decrease of magnetic moments of the neighboring Ni atoms than Al. This corresponds to the larger structural changes in the configuration with substitutional Si which is obvious from the left-hand part of the Figure 8b,c.

In case of the structure GB-Ni_119_Si_2_^(i)^+Va^L3^ (Figure 9d), we can find again two couples of curves with similar variations corresponding to planes with (the red curve with circles and blue with squares) and without (the green curve with diamonds and violet with triangles) the impurity. The atomic positions in this structure have also changed significantly due to the relaxation. As the silicon atom in the vicinity of the vacancy left its position at the grain boundary, the layout of magnetic moments became asymmetric with respect to the GB. Nevertheless, the distance of the Ni atoms from the impurity plays the dominant role also here. At the GB without a vacancy, the magnetic moment of Ni atoms increases from 0.35 to 0.45 μ_B_ with the increasing distance of Ni atom from the Si impurity from 2.18 to 2.46 Å. For atoms in the distance of 2.93 Å and further, the magnetic moments reach the values of 0.61 μ_B_ and higher converging to the bulk value. At the GB with the vacancy, the magnetic moments reveal the same trend as at the GB without vacancy. They change from 0.41 to 0.58 μ_B_ for the distances from 2.26 to 2.80 Å. The Ni atoms placed further from Si reach the magnetic moments of 0.61 μ_B_ and higher. The Ni atoms located simultaneously at the GB and in the vicinity of the Si atom possess low magnetic moments despite of the fact that they are placed in the region with a larger free volume causing in general an increase of the magnetic moment. This demonstrates the dominating effect of the impurity on the magnetic moments. This effect could also be proved by the fact that the Ni atoms placed at different GBs (with and without Va) but having the same distance from impurity exhibit also similar magnetic moments. For example, the magnetic moments of 0.41 and 0.52 μ_B_ are reached at the distances of 2.26 and 2.52 Å from Si atom in the layer with a vacancy. For comparison, at the GB without a vacancy, the magnetic moments of 0.41 and 0.45 μ_B_ are reached at the distances of 2.28 and 2.46 Å from the Si atom. Magnetic moments of interstitial Si atoms are identical within the computational error. The asymmetrical behavior of the blue line with squares in the center of Figure 9d is caused by the presence of the shifted impurity in the neighboring plane. This impurity gets to the vicinity of the Ni atom in the 2nd plane on the right-hand side of central part of the blue line with squares in this figure, which causes the decrease of the magnetic moment of Ni.

The curves in Figure 9d reveal a larger scatter at the GB with vacancy as its free space enables the movement of atoms and, consequently, the change of the distances between Ni atoms and interstitial Si.

It can be concluded that in case of all structures, the distance between the Ni atom and an impurity has the dominating effect on the magnetic moments of Ni. The effect of the vacancy is due to the mediated migration of atoms.

Furthermore, Figure 9b–d also provide the information about the magnetic moments of the impurities which are −0.017, −0.013 and −0.019 μ_B_ for Al^(s)^, Si^(s)^ and Si^(i)^, respectively. These values are comparable with those found in previously studied materials/configurations in this work. The magnetic moments of impurities in the structure with a GB and without a vacancy are −0.016, −0.011 and −0.021 μ_B_ for Al^(s)^, Si^(s)^ and Si^(i)^. In the bulk material with a vacancy, the magnetic moment of Al^(s)^, Si^(s)^ and Si^(i)^ amounts to −0.019, −0.016 and −0.014 μ_B_, respectively, and for the bulk without a vacancy, it is −0.018, −0.014 and −0.021 μ_B_, respectively. Except for the bulk structure with a vacancy, silicon at the interstitial position has always the lowest magnetic moment and silicon at the substitutional position has always the highest magnetic moment.

In the experimental study [71], a GB configuration close to the twist (001) GB was examined. Those authors found magnetic moment enhancement up to about 100%. On the other hand, our values of the enhancement of magnetic moment are rather small. They amount, in maximum, to about 9% (with respect to the bulk). On the other hand, the magnetic moment of Ni atom situated at the Σ5(210) GB was experimentally determined using a transmission electron microscopy/electron energy loss spectroscopy method [72] to be 0.63 μ_B_. This is essentially the same as the magnetic moment of bulk Ni atom (0.632 μ_B_) calculated in this work. Some lattice defects at the GBs, for example vacancies studied here, might slightly enhance this phenomenon although we still were not able to reach the enhancement reported in [71]. However, other theoretical studies [2,49,57,73,74] also demonstrate that the enhancement of magnetic moments at various GBs in Ni is rather small, if not negative [75] (unfortunately, GBs with other defects than segregated impurities have been studied only rarely up to now). Thus, it is not excluded that such a large enhancement reported in [71] may be a spurious effect. Another questionable issue is the fact that the enhancement of magnetic moment found in [71] extends up to a nearly 100 Å distance from the GB plane whereas most theoretical calculations locate it in the region of about several Å. Thus, further experimental as well as theoretical studies are desirable to shed light on these problems.

There are also some issues connected with non-zero temperature. As the atomistic simulations [76,77] demonstrate, with increasing temperature vacancies become unstable; they delocalize and may lead to instability. Consequently, effective disappearance of the free volume connected with vacancies at temperatures of about 600 K at most may occur. Of course, GBs act as vacancy sinks and the effect described above is in full accordance with this fact.

As it is mentioned in [78] the optimized model of a tensile test on clean GB in *fcc* Ni predicted a structural transformation from the Σ5(210) GB to a Σ11(311) GB. In addition, the ab initio simulations of hydrostatic and uniaxial tensile tests on *fcc* crystals of Ni predicted instabilities of an elastic nature in Ni crystal [79]. Further, in [80] the effect of applied stress on vacancy segregation near the Σ5(210) GB in Ni has been reported with the help of a methodology similar to that in the present paper. Here, we have analyzed more vacancy positions – not only those at the GB plane. The authors of the study [80] suggested that vacancies can be absorbed by the GB under a compressive stress with just an opposite effect for a tensile stress applied perpendicular to the GB. Those predictions, however, are not in agreement with known thermodynamic models. In addition to that, the effect of temperature (vibrations) on the behavior of vacancies was not included. This, of course, could influence the results of those calculations.

## 4. Conclusions

A detailed and systematic first-principles study of substitutional Al^(s)^ and both substitutional Si^(s)^ and interstitial Si^(i)^ impurities in the ferromagnetic *fcc* bulk nickel and at the Σ5(210) GB is presented, including various concentrations and positions of impurities not previously studied. This research was complemented by cases where vacancies have also occurred. In all instances, the structural, energetic and magnetic aspects were investigated.

In the bulk material, the addition of impurities caused an increase of the volume per atom in comparison with elemental Ni. The most significant increase was observed in the structures with the tetrahedral (iT) or octahedral (iO) interstitial Si atom. These configurations nevertheless possess a positive energy of formation related to standard states of pure elements, which makes them unstable. Larger structures with interstitial impurities are more stable than the smaller ones. The structure Ni_120_Si^(iO)^ reveals a higher stability than Ni_120_Si^(iT)^ which reflects the fact that the octahedral site is larger than the tetrahedral one. Introducing the substitutional Al or Si into the material led to structure stabilization. The impurities reduce the magnetic moments of their neighboring Ni atoms and they themselves obtain a small negative induced magnetic moment.

Adding vacancies to the bulk material caused a decrease of the cell volume but an increase of the volume per atom in comparison with the bulk structures without a vacancy. The addition of a vacancy always induced an increase of the total energy of the system or the vacancy disappearance during the recombination with the interstitial Si^(i)^ atom. In case of Ni_59_Al^(s)^+Va and Ni_59_Si^(s)^+Va structures, the minimum-energy configurations are those with the vacancy and impurity being the nearest neighbors. In structures with interstitial silicon, the vacancy is stable only if it is far enough from the silicon atom. The preference of the structures with substitutional impurities is sustained even in the presence of the Va. The presence of a vacancy causes an increase of magnetic moments of neighboring Ni atoms by 0.02–0.04 μ_B_. However, this effect is not as strong as the “impurity-caused-decreasing” effect.

The structures containing a GB and an impurity reveal a scattering in the interlayer distances. The largest distance is found between the GB and the second layer and the lowest one occurs between the second and the third layer. For the next interlayer distances, the difference from calculated data [2,49] was not so large and from the sixth layer on, the interlayer distances closely oscillated around the calculated bulk value. The excess free volume gains the values in the range between 0.11 and 0.34 Å^3^.Å^-2^. In all studied cases, the GBs are unstable with respect to the bulk material in the sequence of GB-Ni_120_Si_2_^(i)^ < GB-Ni_60_ = GB-Ni_120_ < GB-Ni_118_Al_2_^(s)^ < GB-Ni_118_Si_2_^(s)^. The segregation energy of substitutional Al^(s)^ atom and interstitial Si^(i)^ atom is negative which means that both impurities tend to be placed in the neighborhood of GB rather than to stay in the bulk material. The substitutional Si^(s)^ atom prefers to be located in the bulk material. However, this arrangement is less stable than the structure with interstitial Si^(i)^. For the structures where the impurity is located at grain boundaries, the “magnetic-moment-decreasing” effect of impurity is suppressing the “magnetic-moment-increasing” effect of the grain boundary. The largest decrease in magnetic moment near to the impurity can be observed in the GB-Ni_120_Si_2_^(i)^ structure.

In case of the structures with vacancies and GBs, the introduction of a vacancy causes changes in positions of atoms, which tend to occupy the free space provided by a vacancy. However, the vacancies in these structures are preserved. For GB-Ni_119_+Va, GB-Ni_117_Al_2_^(s)^+Va and GB-Ni_117_Si_2_^(s)^+Va (GB-Ni_119_Si_2_^(i)^+Va), the configuration with vacancy in layer 2 (layer 3) reveals the highest decrease of the volume and the lowest but positive both energy of vacancy formation and the energy of formation with respect to the standard element reference states. This means that the largest decrease in volume corresponds to the most easily but still unfavorably formed vacancy and to the most stable arrangement of structure with vacancy in a given position. However, as all configurations of structures possess the positive values of the energy of formation they must be considered as unstable at 0 K. Nevertheless, these structures can be stabilized at higher temperatures. In such a case, we expect the Si^(i)^ to be more stable than Si^(s)^. One of the contributions to this instability is the vacancy formation energy, which ranges from 0.25 to 0.62 eV.Va^−1^.

Analyzing the binding energies between the defects, it was found that the binding energy *E^b^(X;Va)* (where X stands for segregated Al or Si) reaches the negative values, which can be interpreted as the tendency to the binding between vacancy and impurity. The *E^b^(GB;Va)* is −0.849 eV for the most stable configuration GB-Ni_119_+Va^L2^. The binding energies between one defect and the remaining couple of defects, *E^b^(Va;GB+X)* and *E^b^(X;GB+Va)*, were analyzed too. It was found that the vacancy tends to stay together with the impurity and GB. The highest released binding energy *E^b^(Va;GB+X)* corresponds to the structure GB-Ni_117_Si_2_^(s)^+Va^L2^ (−1.18 eV) and the other values are also reasonable (more than −0.8 eV). Additionally, the impurities prefer to stay in the neighborhood of the couple GB+Va which is related to the negative binding energy *E^b^(X;GB+Va).* In particular, the tendency of interstitial Si to move from the bulk to a GB with a vacancy is very strong (−8.98 eV). The effects of how two defects interact in the presence of the third one were also investigated. In all studied cases, the interaction is associated with negative (the defects tend to stay together) or negligible energy effects (their interaction neither preferred nor rejected). Considering the binding energies of the triple defect from single defects *E^b^(X;GB;Va)* or couple of defects E^b^(X;GB;Va)*, it was again concluded that the defects tend to stay together except for the case of combining the couple of defects in structure GB-Ni_119_Si_2_^(i)^+Va^L3^.

The theoretical total energy $E_{GB+X^{\left(s/i\right)}+Va}^{theo}$ of studied structures calculated from separated contributions was also evaluated. The lowest value (−330.900 and −324.873 eV.cell^−1^) corresponds to the GB-Ni_120_Si_2_^(i)^ and GB-Ni_119_Si_2_^(i)^+Va^L3^ structure. The difference between the directly calculated and the theoretical energy value $E^{diff}$ was negative for all studied structures with GB and impurity. It indicates that for these structures the directly calculated energy could contain some stabilizing interactions in comparison to the theoretical energy obtained from separated contributions. However, this can be also caused by the fact that the contributions of particular defects and their combinations were calculated from structures at equilibrium volumes which differ from equilibrium volumes of the final structures.

The presence of a vacancy at GB induced changes in the behavior of impurities with respect to segregation—all studied impurities prefer to segregate. When the vacancy is present in both structures (at the GB and in the bulk material) or in the bulk material, the segregation becomes less advantageous and in case of the Si^(s)^ even unfavorable. The strongest tendency to segregation is observed in structures with substitutional impurities and without vacancies.

The GBs and vacancies cause an increase of magnetic moments, nevertheless the impurities, having induced magnetic moments of the opposite direction than Ni atoms, exhibit the dominating decreasing effect on the magnetism of Ni atoms.

Let us note that the segregation energy itself (calculated at 0 K) cannot completely describe the solute segregation but very well characterizes the tendency of the solute to segregate at a grain boundary [1]. Nevertheless, as it follows from Langmuir–McLean segregation isotherm, the segregation energy is an important controlling parameter of the temperature dependence of grain boundary segregation and this constitutes the main point of our paper. The problem of entropy is substantial as it modifies the solute concentration at the boundary [81]. Despite the crucial importance of entropy, any information about the segregation energy—as a part of the total driving force for segregation—is also valuable for experimentalists.

## Figures and Tables

**Table 1:** Equilibrium properties of the bulk material of all three elements studied and *fcc* Ni containing impurities. Here, *a*, *b* and *c* stand for lattice parameters, *E^f^* is the energy of formation related to the standard element reference states: ferromagnetic *fcc* Ni, nonmagnetic *fcc* Al and nonmagnetic Si in the diamond structure (Equation (1)). *μ*_Ni_ stands for the magnetic moment per atom obtained by averaging over all Ni atoms in the structure and *μ*_Ni,NN_ denotes the magnetic moment of Ni atom that is the nearest neighbor of the impurity. Superscripts (s) and (iT) or (iO) mark the substitutional or interstitial impurity position. The *fcc* supercell lattice parameters obtained from literature were derived from the lattice parameters of *fcc* Ni unit cell *a* = 3.498 Å [30] and *a* = 3.5138 Å [31].

Configuration	*a*	*b*	*c*	*V_at_*	*E^f^*	*μ*_Ni_	*μ*_Ni,NN_	Reference
	(Å)	(Å)	(Å)	(Å^3^)	(eV.atom^−1^)	(μ_B_)	(μ_B_)	
Si	5.469	5.469	5.469	20.443	0	-	-	This work
Al	4.040	4.040	4.040	16.481	0	-	-	This work
Ni_60_	7.866	7.866	10.552	10.882	0	0.632	-	This work
	7.824	7.824	10.497	10.710	-	0.628	-	[32]
	7.821	7.821	10.494	10.669	-	0.607	-	[30] ^a^
	7.857	7.857	10.541	10.846	-	-	-	[31] ^a^
Ni_59_Al^(s)^	7.872	7.872	10.562	10.909	−0.026 **	0.608	0.525	This work
	-	-	-	10.735	-	-	0.528	[32]
	-	-	-	-	−0.029	-	-	[33,34] ^b^
Ni_59_Si^(s)^	7.866	7.866	10.550	10.878	−0.027 **	0.624	0.499	This work
	-	-	-	10.699	-	-	0.484	[32]
	-	-	-	-	−0.031	-	-	[33,34] ^b^
Ni_60_Si^(iT)^ *	7.945	7.929	10.654	11.003	0.024	0.579	0.278	This work
Ni_120_Si^(iT)^	7.899	7.899	21.245	10.956	0.020	0.600	0.251	This work
Ni_60_Si^(iO)^ *	7.938	7.938	10.670	11.021	0.026	0.598	0.302	This work
Ni_120_Si^(iO)^	7.903	7.903	21.218	10.951	0.014 **	0.623	0.302	This work

* The interstitial impurity migrated away from the ideal tetrahedral (octahedral) position. ** From these values, the energies of entering of one impurity into the pure *fcc* nickel ($E^{f}$ *(X)*) used in Equation (20) were evaluated by multiplying these values by the total number of atoms in the structure. These values are *E^f^(Al*^(s)^*)* = −1.574 eV.X^−1^, *E^f^(Si*^(s)^*)* = −1.608 eV.X^−1^ and *E^f^(Si*^(i)^*)* = −1.669 eV.X^−1^. ^a^ The data were calculated for Ni_4_ unit cell. ^b^ The formation energy was obtained by the extrapolation of the formation energies for Ni, Ni_3_Al, Ni_3_Si by Open Quantum Mechanics Database tools.

**Table 2:** Equilibrium properties of bulk material with vacancies (both elemental *fcc* Ni and *fcc* Ni containing impurities). Va means one vacancy in the structure, *D* denotes the equilibrium distance of Va from the impurity, *a*, *b* and *c* stand for lattice parameters, *V_at_* denotes the volume per atom, *E^f^* is the energy of formation related to the ground-state of elemental ferromagnetic *fcc* Ni, *fcc* Al and diamond Si. *E^f^(Va)* is the energy of vacancy formation and *E^b^(X;Va)* is the binding energy between impurity X and vacancy. The values written in bold correspond to the most stable configuration.

Config.	*D* (Å)	*a* (Å)	*b* (Å)	*c* (Å)	*V_at_* (Å^3^)	*E^f^* (eV.atom^−1^)	*E^f^(Va)* (eV.Va^−1^)	*E^b^(X;Va)* (eV.X^−1^Va^−1^)
Ni_59_+Va	-	7.8496	7.8497	10.5332	11.0003	0.0239	1.4101	-
Ni_58_Al^(s)^+Va	**2.4594**	**7.8541**	**7.8652**	**10.5256**	**11.0206**	**−0.0035**	**1.3664**	**−0.0451**
	3.5249	7.8566	7.8561	10.5375	11.0236	−0.0022	1.4431	0.0316
	4.2946	7.8595	7.8607	10.5340	11.0307	−0.0023	1.4372	0.0257
	4.9448	7.8562	7.8615	10.5326	11.0255	−0.0029	1.4018	−0.0097
Ni_58_Si^(s)^+Va	**2.4263**	**7.8489**	**7.8409**	**10.5318**	**10.9856**	**−0.0052**	**1.3005**	**−0.1110**
	3.5024	7.8490	7.8493	10.5229	10.9883	−0.0036	1.3949	−0.0166
	4.2894	7.8485	7.8495	10.5259	10.9910	−0.0030	1.4303	0.0188
	4.9468	7.8462	7.8470	10.5296	10.9881	−0.0034	1.4067	−0.0048
Ni_59_Si^(iT)^+Va (Ni_59_Si^(s)^*)	1.5092 *	7.8628	7.8630	10.5499	10.8050	−0.0269	−3.0488 *	--- *
	2.9128 *	7.8630	7.8628	10.5500	10.8050	−0.0269	−3.0488 *	--- *
	3.8100 *	7.8630	7.8630	10.5498	10.8050	−0.0269	−3.0492 *	--- *
	4.5578 *	7.8643	7.8645	10.5406	10.8654	−0.0269	−3.0485 *	--- *
	5.2036 *	7.8630	7.8628	10.5499	10.8709	−0.0269	−3.0488 *	--- *
Ni_119_Si^(iT)^+Va (Ni_119_Si^(s)^*)	1.5233 *	7.8639	7.8644	21.0950	10.8718	−0.0136	−4.0395 *	--- *
	2.9169 *^,a^	8.2455	10.8490	14.9017	11.1086	−0.0141	−4.1043 *	--- *
	4.5700 *^,a^	9.5257	9.2313	14.8911	10.9022	0.0098	−1.2291 *	--- *
	**5.1815** ^b^	**7.8916**	**7.8899**	**21.2043**	**11.0021**	**0.0244**	**0.5153**	**−0.1578**
Ni_59_Si^(iO)^+Va (Ni_59_Si^(s)^*)	1.7603 *	7.8632	7.8633	10.5498	10.8717	−0.0271	−3.2177 *	--- *
	**3.4306 ^c^**	**7.9078**	**7.9159**	**10.6642**	**11.1259**	**0.0461**	**1.1772**	**−0.2343**
	3.9362 *	7.8629	7.8629	10.5494	10.8703	−0.0271	−3.2181 *	--- *
	5.2802 *	7.8625	7.8630	10.5499	10.8704	−0.0271	−3.2192 *	--- *
Ni_119_Si^(iO)^+Va (Ni_119_Si^(s)^*)	1.7436 *	7.8631	7.8631	21.1068	10.8749	−0.0138	−3.3306 *	--- *
	**3.1287**	**7.9000**	**7.8963**	**21.2041**	**11.0227**	**0.0257**	**1.4128**	0.0012 **
	3.9332 *	7.8623	7.8629	21.1038	10.8720	−0.0138	−3.3306 *	--- *
	5.2770 *	7.8669	7.8669	21.1127	10.8886	−0.0268	−4.8852 *	--- *

* The structure in parentheses denotes the final equilibrium arrangement after the relaxation and the *D* values correspond to the distances before relaxation. *E^f^* corresponds to the energy of formation of Ni_59_Si^(s)^ or Ni_119_Si^(s)^_._ In this case, *E^f^(Va)* is the energy difference between structures with interstitial and substitutional Si and *E^b^(X;Va)* cannot be evaluated as Va disappeared during the structure relaxation. ** In case of the structure Ni_119_Si^(iO)^+Va, the value of *E^b^(X;Va)* was calculated with respect to structures Ni_59_+Va and Ni_120_Si^(iO)^. Hence, the value −0.2343 eV.X^−1^Va^−1^ should be considered more reliable as it was calculated using the energies of structures with comparable size. ^a^ The shape of the cell has changed and the equilibrium angles are not *α* = *β* = *γ* = 90.00°. ^b^ The value of *D* before relaxation was 3.8336 Å. ^c^ The value of *D* before relaxation was 3.0489 Å.

**Table 3:** Properties of structures with clean and impurity-segregated GBs (equilibrium *b/a* parameter, volume per atom *V_at_*, excess free volume *V^f^* and GB energy *γ_GB_*) obtained by different methods of relaxation. Method 1 is the automatic full relaxation. Method 2 keeps the two lattice parameters (in the plane of the GB) fixed and the lattice parameter perpendicular to the GB is changed by the simultaneous relaxation of atomic positions. The equilibrium structures were determined with respect to (a) minimum energy (Method 2a) or (b) the zero stress along the dimension perpendicular to the GB (Method 2b). Here, *b* is the supercell lattice constant perpendicular to the GB plane and *a* is the lattice constant in the plane of the GB (see Figure 1b).

**Method of relax.**	**GB-Ni_60_**				**GB-Ni_120_**							
	***b*/*a***	***V_at_*** **(Å ^3^)**	***V^f^*** **(Å^3^.Å^−2^)**	***γ_GB_*** **(J.m^−2^)**	***b/a***	***V_at_*** **(Å^3^)**	***V^f^*** **(Å^3^.Å^−2^)**	***γ_GB_*** **(J.m^−2^)**				
1	3.13	11.12	0.26	1.29 1.23 [2]	3.13	11.12	0.26	1.29				
2a	3.09	11.20	0.34	1.31 1.43 * [50]	3.09	11.20	0.34	1.30				
2b	3.09	11.20	0.34	1.31	3.09	11.21	0.35	1.30				
**Method of relax.**	**GB-Ni_118_Al_2_^(s)^**				**GB-Ni_118_Si_2_^(s)^**				**GB-Ni_120_Si_2_^(i)^**			
	***b/a***	***V_at_*** **(Å ^3^)**	***V^f^*** **(Å^3^.Å^−2^)**	***γ_GB_*** **(J.m^−2^)**	***b/a***	***V_at_*** **(Å ^3^)**	***V^f^*** **(Å^3^.Å^−2^)**	***γ_GB_*** **(J.m^−2^)**	***b/a***	***V_at_*** **(Å ^3^)**	***V^f^*** **(Å^3^.Å^−2^)**	***γ_GB_*** **(J.m^−2^)**
1	3.11	11.14	0.26	2.50	3.12	11.11	0.26	2.71	3.15	11.10	0.11	0.64
2a	3.08	11.22	0.29	2.53	3.08	11.19	0.28	2.75	3.13	11.13	0.13	0.65
2b	3.09	11.23	0.29	2.53	3.08	11.19	0.28	2.75	3.13	11.13	0.14	0.65

* The computational cell was GB-Ni_40_.

**Table 4:** Segregation energies obtained by different methods of relaxation. Here eV.X^−1^ stands for eV per atom of impurity.

$E^{seg}\left(X\right)\;(\mathrm{eV}.X^{-1})$			
Structure	GB-Ni_118_Al_2_^(s)^	GB-Ni_118_Si_2_^(s)^	GB-Ni_120_Si_2_^(i)^
Method of relaxation			
1	−0.144	0.222	−0.258
	−0.22 * [2]		−0.76 * [2]
	0.05 * [8]		−0.71 * [8]
2a	−0.132	0.252	−0.281
	−0.19 * [50]		−0.83 * [50]
2b	−0.132	0.252	−0.275

* The values of segregation energy are not directly comparable with our results because of various sizes of unit cells and number of impurities. In Reference [2], the unit cells are GB-Ni_56_Al_4_^(s)^ and GB-Ni_60_Si_4_^(i)^; Reference [8] deals with the unit cells GB-Ni_79_Al_1_^(s)^ and GB-Ni_80_Si_1_^(i)^ and in Reference [50], the unit cell corresponds to GB-Ni_36_Al_4_^(s)^ and GB-Ni_40_Si_4_^(i)^.

**Table 5:** Magnetic moment of Ni atoms *μ_at_* in the neighborhood of impurity X. *R* stands for the distance from impurity.

Structure	Plane	*R* (Å)	*μ_at_* (μ_B_)	Layer
GB-Ni_118_Al_2_^(s)^ GB (Ni,X)	clean	2.586	0.528	3
	clean	2.599	0.581	2
	with X	2.634	0.588	2
	with X	2.639	0.554	4
GB-Ni_118_Si_2_^(s)^ GB (Ni,X)	clean	2.504	0.483	3
	clean	2.588	0.572	2
	with X	2.592	0.563	2
	with X	2.645	0.552	4
GB-Ni_120_Si_2_^(i)^ GB (Ni,X)	with X	2.177	0.357	3
	clean	2.278	0.429	2
	with X	2.312	0.420	GB (Ni,X)
	clean	2.444	0.453	GB (Ni,X)

**Table 6:** Energy and volume effects associated with vacancy formation in various layers of GB-Ni_119_+Va, GB-Ni_117_Al_2_^(s)^+Va, GB-Ni_117_Si_2_^(s)^+Va and GB-Ni_119_Si_2_^(i)^+Va structure. The quantity *E^f^(Va)* is defined as the formation energy of the vacancy. *V^f^* is the change of volume of cell associated with the formation of vacancy and it is defined as the difference of the volume of the structure with and without a vacancy. The values written in bold correspond to the most stable configurations.

Structure	GB-Ni_119_+Va		GB-Ni_117_Al_2_^(s)^+Va		GB-Ni_117_Si_2_^(s)^+Va		GB-Ni_119_Si_2_^(i)^+Va	
Layer with Va	*E^f^*(*Va*) (eV.Va^−1^)	*V^f^* (Å^3^.Va^−1^)	*E^f^*(*Va*) (eV.Va^−1^)	*V^f^* (Å^3^.Va^−1^)	*E^f^*(*Va*) (eV.Va^−1^)	*V^f^* (Å^3^.Va^−1^)	*E^f^*(*Va*) (eV.Va^−1^)	*V^f^* (Å^3^.Va^−1^)
GB	1.5727	−1.2460	1.5808	6.3510	1.4961	2.6906	1.4304	−5.6628
2	**0.5791**	**−9.7127**	**0.6169**	**−3.3396**	**0.2466**	**−5.7481**	0.9106	−10.8557
3	1.2866	−4.0505	1.2833	3.2737	1.2105	−1.3837	**0.5746**	**−14.3407**
4	1.3088	−4.9483	1.3428	1.5724	1.1510	−5.5262	1.2894	−8.8176
5	1.2874	−4.7500	1.3190	1.8658	1.2819	−0.4996	1.3253	−9.1865

**Table 7:** Energy of formation of structures GB-Ni_119_+Va, GB-Ni_117_Al_2_^(s)^+Va, GB-Ni_117_Si_2_^(s)^+Va and GB-Ni_119_Si_2_^(i)^+Va with three defects (GB, impurity, vacancy) for a vacancy in various layers. The quantity $E^{f}\left(GB-Ni_{m}Al_{n}Si_{o}+Va\right)$ is defined with respect to the standard element reference states. The values written in bold correspond to the lowest energy configuration.

Structure	GB-Ni_119_+Va	GB-Ni_117_Al_2_^(s)^+Va	GB-Ni_117_Si_2_^(s)^+Va	GB-Ni_119_Si_2_^(i)^+Va
Layer with Va	$E^{f}\left(GB-Ni_{m}Al_{n}Si_{o}+Va\right)$ (eV.cell^−1^)			
GB	10.33	6.94	7.52	6.50
2	**9.34**	**5.97**	**6.27**	5.98
3	10.05	6.64	7.24	**5.64**
4	10.07	6.70	7.18	6.36
5	10.05	6.68	7.31	6.39

**Table 8:** The energetic characteristics of interaction of three defects: the binding energy of a vacancy to the couple of GB+X (*E^b^(Va;GB+X)*), the binding energy of an impurity to the couple of GB+Va (*E^b^(X*;*GB+Va)*), the vacancy-impurity binding energy at the GB for substitutional or interstitial impurity (*E^b^(GB+X*^(s/i)^;*GB+Va)*), the vacancy-GB binding energy in the presence of substitutional or interstitial impurity (*E^b^(X*^(s/i*)*^*+Va;X^(^*^s/i*)*^*+GB)*), the X-GB binding energy in the presence of Va (*E^b^(Va+X^(^*^s/i*)*^*;Va+GB)*) for GB-Ni_117_Al_2_^(s)^+Va^L2^, GB-Ni_117_Si_2_^(s)^+Va^L2^ and GB-Ni_119_Si_2_^(i)^+Va^L3^ structures.

Structure	*E^b^(Va;GB+X)*	*E^b^(X;GB+Va)*	*E^b^(GB+X;GB+Va)*	*E^b^(X+Va;X+GB)*	*E^b^(Va+X;Va+GB)*
	(eV)	(eV)	(eV.X^−1^Va^−1^)	(eV.Va^−1^GB^−1^)	(eV.X^−1^GB^−1^)
GB-Ni_117_Al_2_^(s)^+Va^L2^	−0.81	−0.10	0.04	−0.75	−0.04
GB-Ni_117_Si_2_^(s)^+Va^L2^	−1.18	−0.11	−0.33	−1.05	0.02
GB-Ni_119_Si_2_^(i)^+Va^L3^	−0.85	−8.98	0.00	−0.84	−3.52

**Table 9:** The formation energy of the vacancy-impurity couple defect at GB (*E^f^(X+Va)*), the binding energy of the triple defect with respect to the total energy of three single defects (GB, X, Va) (*E^b^(X;GB;Va)*) and the binding energy of triple defect with respect to the total energy of three couple defects (GB+X, X+Va, Va+GB) (E^b^(X;GB;Va)*) for GB-Ni_117_Al_2_^(s)^+Va^L2^, GB-Ni_117_Si_2_^(s)^+Va^L2^ and GB-Ni_119_Si_2_^(i)^+Va^L3^ structure.

Structure	*E^f^(X+Va)*	*E^b^(X;GB;Va)*	E^b^(X;GB;Va)*
	(eV.X^−1^Va^−1^)	(eV.X^−1^Va^−1^GB^−1^)	(eV.X^−1^Va^−1^GB^−1^)
GB-Ni_117_Al_2_^(s)^+Va^L2^	−1.08	−0.92	0.08
GB-Ni_117_Si_2_^(s)^+Va^L2^	−1.12	−0.92	−0.22
GB-Ni_119_Si_2_^(i)^+Va^L3^	−1.27	−4.35	0.31

**Table 10:** Theoretical total energies of studied structures $E_{GB+X^{\left(s/i\right)}+Va}^{theo}$ and the difference between the total energies calculated ab initio and theoretical total energies (calculated from the particular contributions) $E^{diff}$ for Ni_59_Al^(s)^, Ni_59_Si^(s)^, Ni_120_Si^(iO)^, Ni_59_+Va, Ni_58_Al^(s)^+Va, Ni_58_Si^(s)^+Va, Ni_59_Si^(iO)^+Va, GB-Ni_120_, GB-Ni_118_Al_2_^(s)^, GB-Ni_118_Si_2_^(s)^, GB-Ni_120_Si_2_^(i)^, GB-Ni_119_+Va^L2^, GB-Ni_117_Al_2_^(s)^+Va^L2^, GB-Ni_117_Si_2_^(s)^+Va^L2^ and GB-Ni_119_Si_2_^(i)^+Va^L3^ structures. In case of the structures with GB, the energies $E_{GB+X^{\left(s/i\right)}+Va}^{theo}$ are related to one GB and X. From this reason, these energies are comparable with those of structures without GB.

Structure	$E_{GB+X^{\left(s/i\right)}+Va}^{theo}$	$E^{diff}$	Structure	$E_{GB+X^{\left(s/i\right)}+Va}^{theo}$	$E^{diff}$	
	(eV.cell^−1^)	(eV.cell^−1^)		(eV.cell^*−1^)	(eV.cell^*−1^)	(kJ.mol of atoms^−1^)
---	---	---	GB-Ni_120_	−323.654	0.000	0.000
Ni_59_Al^(s)^	−327.888	0.000	GB-Ni_118_Al_2_^(s)^	−323.596	−0.037	−0.060
Ni_59_Si^(s)^	−329.598	0.000	GB-Ni_118_Si_2_^(s)^	−324.935	−0.043	−0.070
Ni_120_Si^(iO)^	−659.820	0.000	GB-Ni_120_Si_2_^(i)^	−330.900	−0.025	−0.039
Ni_59_+Va	−321.155	0.000	GB-Ni_119_+Va^L2^	−317.601	−0.007	−0.011
Ni_58_Al^(s)^+Va	−321.054	0.000	GB-Ni_117_Al_2_^(s)^+Va^L2^	−317.536	−0.014	−0.022
Ni_58_Si^(s)^+Va	−322.830	0.000	GB-Ni_117_Si_2_^(s)^+Va^L2^	−319.258	−0.006	−0.010
Ni_59_Si^(iO)^+Va	−325.222	0.000	GB-Ni_119_Si_2_^(i)^+Va^L3^	−324.873	−0.011	−0.017

* denotes the cell consisting of one GB, one impurity and one vacancy.

**Table 11:** Segregation energies of impurity segregating (**a**) from the bulk to GB (*E^seg^(X)*, Equation (7)); (**b**) from the bulk to GB with Va ($E_{Va^{GB}}^{seg}\left(X\right)$, Equation (22)); (**c**) from the bulk with Va to GB ($E_{Va^{bulk}}^{seg}\left(X\right)$, Equation (23)) and (**d**) from the bulk with Va to GB with Va ($E_{Va^{bulk};Va^{GB}}^{seg}\left(X\right)$, Equation (24)) for GB-Ni_117_Al_2_^(s)^+Va^L2^, GB-Ni_117_Si_2_^(s)^+Va^L2^ and GB-Ni_119_Si_2_^(i)^+Va^L3^ structure.

Structure	$E^{seg}\left(X\right)$	$E_{Va^{GB}}^{seg}\left(X\right)$	$E_{Va^{bulk}}^{seg}\left(X\right)$	$E_{Va^{bulk};Va^{GB}}^{seg}\left(X\right)$
	(eV.X^−1^)	(eV.X^−1^)	(eV.X^−1^)	(eV.X^−1^)
GB-Ni_117_Al_2_^(s)^+Va^L2^	−0.125	−0.104	−0.080	−0.042
GB-Ni_117_Si_2_^(s)^+Va^L2^	0.241	−0.108	0.352	0.019
GB-Ni_119_Si_2_^(i)^+Va^L3^	−3.518	−0.260	−0.128	−0.132

## Supplementary Materials

The following are available online at https://www.mdpi.com/2079-4991/10/4/691/s1. Table S1: Data depicted in Figure 4: the equilibrium interlayer distances *D* for structures GB-Ni_60_, GB-Ni_118_Al_2_^(s)^, GB-Ni_118_Si_2_^(s)^ and GB-Ni_120_Si_2_^(i)^ obtained from the automatic relaxation. Here, the interlayer distance of type 2/3 stands for the distance between the 2nd and 3rd layer under the assumption that the 1st layer is the layer of GB. Further, Ni,Ni and X,Ni stand for the clean plane and the plane with impurity X = Al, Si, respectively.
