# Supplementary material for: The Effect of Vacancies on Grain Boundary Segregation in Ferromagnetic fcc Ni
Source: Nanomaterials (Basel). 2020 Apr 6;10(4):691. doi: 10.3390/nano10040691 (PMC7221896; doi:10.3390/nano10040691)
Supplement: Supplementary file 1 [file nanomaterials-10-00691-s001.pdf]

Supplementary Material:

Table S1. Data depicted in Figure 4: the equilibrium interlayer distances *D* for structures GB-Ni_{60}, GB-$Ni_{118}Al_2^{(s)}$, GB-$Ni_{118}Si_2^{(s)}$ and GB-$Ni_{120}Si_2^{(i)}$ obtained from the automatic relaxation. Here, the interlayer distance of type 2/3 stands for the distance between the 2nd and 3rd layer under the assumption that the 1st layer is the layer of GB. Further, Ni,Ni and X,Ni stand for the clean plane and the plane with impurity X = Al, Si, respectively.

Type of interlayer distance	Interlayer distance *D* (Å)							
	GB-Ni_{120}	GB-Ni_{60}	GB-$Ni_{118}Al_2^{(s)}$		GB-$Ni_{118}Si_2^{(s)}$		GB-$Ni_{120}Si_2^{(i)}$	
	Ni,Ni	Ni,Ni	Ni,Ni	Al,Ni	Ni,Ni	Si,Ni	Ni,Ni	Si,Ni
GB/2	1.1078	1.1078	1.1119	1.1135	1.1072	1.1070	1.1381	1.1407
2/3	0.5743	0.5743	0.5668	0.5931	0.5308	0.5950	0.6250	0.7024
3/4	0.8584	0.8584	0.8579	0.8400	0.9057	0.8357	0.8742	0.7554
4/5	0.8248	0.8248	0.8349	0.8289	0.8273	0.8332	0.8000	0.8303
5/6	0.7512	0.7512	0.7502	0.7538	0.7438	0.7543	0.7603	0.7885
6/7	0.8053	0.8053	0.8130	0.8008	0.8132	0.8038	0.8060	0.7763
7/8	0.8041	0.8041	0.8019	0.8038	0.8076	0.8034	0.7971	0.7982
8/8	0.7672	0.7672	0.7631	0.7685	0.7645	0.7716	0.7733	0.7899
7/8	0.8041	0.8041	0.8073	0.8038	0.8076	0.8077	0.7971	0.7982
6/7	0.8053	0.8053	0.8000	0.8008	0.8132	0.8006	0.8060	0.7763
5/6	0.7512	0.7512	0.7439	0.7538	0.7438	0.7570	0.7603	0.7885
4/5	0.8248	0.8248	0.8216	0.8289	0.8273	0.8294	0.8000	0.8303
3/4	0.8584	0.8584	0.8523	0.8400	0.9057	0.8509	0.8742	0.7554
2/3	0.5743	0.5743	0.6007	0.5931	0.5309	0.5787	0.6250	0.7024
GB/2	1.1078	1.1078	1.1107	1.1135	1.1073	1.1071	1.1401	1.1433
